# Myogenesis in the sea urchin embryo: the molecular fingerprint of the myoblast precursors

**DOI:** 10.1186/2041-9139-4-33

**Published:** 2013-12-02

**Authors:** Carmen Andrikou, Edmondo Iovene, Francesca Rizzo, Paola Oliveri, Maria Ina Arnone

**Affiliations:** 1 Cellular and Developmental Biology, Stazione Zoologica Anton Dohrn, Napoli 80121, Italy; 2 Genetic, Evolution and Environment Department, University College London, London WC1E 6BT, UK; 3 Current address: Laboratory of Molecular Medicine and Genomics, Department of Medicine and Surgery, University of Salerno, Salerno 84081, Italy

**Keywords:** Mesoderm, Muscle, Myogenic regulatory factor, Regulatory state, Myosin heavy chain, Forkhead, MyoD, Cooption

## Abstract

**Background:**

In sea urchin larvae the circumesophageal fibers form a prominent muscle system of mesodermal origin. Although the morphology and later development of this muscle system has been well-described, little is known about the molecular signature of these cells or their precise origin in the early embryo. As an invertebrate deuterostome that is more closely related to the vertebrates than other commonly used model systems in myogenesis, the sea urchin fills an important phylogenetic gap and provides a unique perspective on the evolution of muscle cell development.

**Results:**

Here, we present a comprehensive description of the development of the sea urchin larval circumesophageal muscle lineage beginning with its mesodermal origin using high-resolution localization of the expression of several myogenic transcriptional regulators and differentiation genes. A few myoblasts are bilaterally distributed at the oral vegetal side of the tip of the archenteron and first appear at the late gastrula stage. The expression of the differentiation genes *Myosin Heavy Chain*, *Tropomyosin I* and *II*, as well as the regulatory genes *MyoD2, FoxF, FoxC, FoxL1, Myocardin, Twist,* and *Tbx6* uniquely identify these cells. Interestingly, evolutionarily conserved myogenic factors such as *Mef2*, *MyoR* and *Six1/2* are not expressed in sea urchin myoblasts but are found in other mesodermal domains of the tip of the archenteron. The regulatory states of these domains were characterized in detail. Moreover, using a combinatorial analysis of gene expression we followed the development of the *FoxF/FoxC* positive cells from the onset of expression to the end of gastrulation. Our data allowed us to build a complete map of the Non-Skeletogenic Mesoderm at the very early gastrula stage, in which specific molecular signatures identify the precursors of different cell types. Among them, a small group of cells within the *FoxY* domain, which also express *FoxC* and *SoxE,* have been identified as plausible myoblast precursors. Together, these data support a very early gastrula stage segregation of the myogenic lineage.

**Conclusions:**

From this analysis, we are able to precisely define the regulatory and differentiation signatures of the circumesophageal muscle in the sea urchin embryo. Our findings have important implications in understanding the evolution of development of the muscle cell lineage at the molecular level. The data presented here suggest a high level of conservation of the myogenic specification mechanisms across wide phylogenetic distances, but also reveal clear cases of gene cooption.

## Background

Muscle development is a highly regulated process that relies on inductive signals to activate a cascade of regulatory events that direct cellular differentiation [1-3]. The molecular events that underlie myogenesis are well documented in several divergent species (for example, mouse and fly) [4,5]. These mechanisms have served as a paradigm for transcriptional regulation since the discovery of myogenic regulatory factors (MRFs), which are able to convert undifferentiated non-mesodermal cells into muscle-like cells [6]. The control mechanism for muscle gene activation appears to be highly conserved, as MRFs from both the sea urchin and the nematode *C. elegans* can efficiently activate myogenesis in 10 T1/2 cells [7,8]. Other transcription factors with an evolutionary conserved role in orchestrating myogenesis are members of the *Forkhead* (Fox) [9,10] and *Sry*-related HMG box (Sox) families [11,12], members of the homeobox *sine oculis* (Six) family [13], the bHLH factors *Twist*[14] and Myogenic Repressor (*MyoR*) [15,16], members of the MADS box family, such as the Myogenic enhancer (*Mef2*) [17,18] and the Serum Response Factor (SRF) *Myocardin*[19,20] and members of the T-box family, *Tbx1* and *Tbx6*[21,22].

Echinoderm larvae have a net of circumesophageal muscles of mesodermal origin that enable swallowing [23]. These are distinguished from another type of endodermally-derived muscle cells that are located in the three myoepithelial sphincters that compartmentalize the archenteron [24,25]. In addition, a third lineage of muscle cells forms paired star-shaped muscles that were recently identified in the ectoderm of the mature Echinoidae plutei, but are absent in the Asteroidea and Holothuroidea larvae [26]. In the sea urchin embryo, the development of circumesophageal muscles has been well characterized from a morphological point of view [23,26]. During gastrulation, Non-skeletogenic mesodermal (NSM) cells delaminate from the coelomic epithelium at the tip of the archenteron. Although most of these cells develop into pigment cells or blastocoelar cells, a small population is committed to differentiate into esophageal muscle cells [23,25,27,28]. During the prism stage, a few cells from each coelomic pouch extend pseudopods toward the outer surface of the esophagus. These cells, known as myoblasts, increase in number and diameter, fuse with each other in the midline of the esophagus and finally form the contractile bands that will surround the esophagus [23,27,29].

Defects in muscle formation caused by perturbations of transcription factors, such as Twist [30] and FoxY [31], or signalling pathways, like Delta/Notch (D/N) [32,33] and Hedgehog (Hh) [34], have been reported in a number of studies. However, only a few homologues of known myogenic regulators in other species have been identified in the sea urchin and their functions in myogenesis remain mostly unknown [7,35]. Moreover, little information exists about the origin, position and molecular identity of the myoblast precursors relative to the other mesodermal cell types in the early stages of development [36].

In this study, we present a thorough description of myogenesis in the sea urchin embryo that includes the identification and characterization of evolutionarily conserved muscle regulatory genes (for example, *Mef2, Twist, MyoR, Tbx6* and *Myocardin)* and terminal differentiation genes (for example, *Myosin heavy chain (MHC), F-actin capping* (*CapZ*) and *Tropomyosin*). These data establish the molecular fingerprint of sea urchin myoblasts. We have also characterized *MyoD1*, previously referred to as *Sum1*[7,37] and another MyoD paralogue, *MyoD2.* Given the expression pattern of these two genes, we suggest that *MyoD1* was co-opted to serve the skeletogenic lineage whilst *MyoD2* acts as an MRF in sea urchin myogenesis. Furthermore, we present a schematic map of the vegetal plate at the very early gastrula stage that illustrates the relative position of the putative myoblast precursors with respect to other NSM cells, including the blastocoelar and pigment cell precursors. This analysis establishes a detailed map of the regulatory state of the NSM at the tip of the late gastrula archenteron.

## Methods

### Animal husbandry and embryo cultures

Adult *Strongylocentrotus purpuratus* were obtained from Patrick Leahy (Kerchoff Marine Laboratory, California Institute of Technology, Pasadena, CA, USA) and housed in circulating sea water aquaria in the Stazione Zoologica Anton Dohrn of Naples. Spawning was induced by vigorous shaking of animals or by intracoelomic injection of 0.5 M KCl. Embryos were cultured at 15°C in Millipore-filtered Mediterranean seawater (MFSW) diluted 9:10 (V:V) in deionized H_2_O. No ethical approval was needed as *Strongylocentrotus purpuratus* is not subject to any animal care regulations.

### Candidate gene search and phylogenetic analysis

Fragments of *Sum1/MyoD1, MyoD2, MyoR2, Twist, Eya, Maf* and *CapZ* were amplified from cDNA and genomic DNA templates by PCR using specific primers (see Additional file 1: Table S1). PCR products were purified and cloned into PcrIItopo (Invitrogen, Carlsberg, CA, USA) according to the manufacturer’s instructions and the identity of inserts confirmed by sequencing. Phylogenetic reconstruction was carried out using the neighbor-joining method, and bootstrap values determined by 1,000 replicates. Homologous sequences were all obtained by database searches using BLASTP and TBLASTX (http://www.ncbi.nlm.nih.gov/BLAST/). Sequences for some Myosin heavy chain (MHC homologs were provided by Patrick Steinmetz [38] (see Additional file 2: Table S2). For phylogenetic analyses, full-length protein sequences were used with the exception of MyoD, in which all sequences were truncated to correspond to the fragment of the sea urchin MyoD2 protein. Trees were also generated using maximum parsimony methods with bootstrap replicates of 1,000 and similar results were obtained. Phylogenetic trees were visualized and edited using Treeview software (http://taxonomy.zoology.gla.ac.uk/rod/treeview.html).

### Whole mount *in situ* hybridization (WMISH)

Embryos and larvae were collected as needed and fixed overnight in 4% paraformaldehyde in 3-(N-morpholino) propanesulfonic acid (MOPS) buffer, washed in MOPS buffer and stored in 70% ethanol until use. *In situ* RNA probe sequences for *FoxY, FoxC, FoxF, FoxL1, Ese, Nanos* and *Gcm* are as previously published (*FoxY*: [39]; *FoxC, FoxL1, FoxF*: [40]; *Ese*: [41]; *Nanos:*[42]; *Gcm:*[39]). *Six1/2, Tbx6, Mef2, SoxE, SoxC, MHC, Tropomyosin1, Tropomyosin2, MYP* and *Myocardin* bacterial clones were picked from the *S. purpuratus* cDNA library available in the laboratory [43,44]. Labeled probes were transcribed from linearized DNA using digoxygenin-11-UTP or fluorescein-12-UTP (Roche, Indianapolis, IN, USA), or labeled with DNP (Mirus, Madison, WI, USA) following kit instructions. For single gene expression, we followed the protocol outlined in [45]. Double fluorescent *in situ* hybridization (FISH) was performed as described [46], with the following modifications. Fixed embryos were washed four times in MOPS buffer, pre-hybridized for 3 hours at 50°C in hybridization buffer and incubated for one week at 50°C with antisense labeled probes, post-hybridized for 3 hours at 50°C and washed four times in MOPS buffer at room temperature. Embryos were then blocked for 30 minutes in fresh 0.5% Perkin Elmer Blocking Reagent (PEBR) in MOPS buffer and incubated overnight with peroxidase-conjugated antibodies at 4°C (Roche, Perkin Elmer 1:1000 dilution). Antibodies were removed with four washes in MOPS buffer, and signal was developed with fluorophore-conjugated tyramide (1:400 reagent diluents, Perkin Elmer). Residual enzyme activity was inhibited via 30-minute incubation in 0.1% hydrogen peroxide followed by four MOPS buffer washes prior to addition and development of the second peroxidase-conjugated antibody. Immunohistochemistry coupled to WMISH was also performed by incubating anti-acetylated tubulin (Sigma-Aldrich, St Louis, MO, USA) antibody together with the first peroxidase conjugated antibody in a dilution 1:250 followed by a second incubation in a 1:1000 dilution of Alexa488 conjugated anti-mouse IgG (Invitrogen, Carlsberg, CA, USA) together with the second peroxidase-conjugated antibody. Embryos were imaged with a Zeiss Axio Imager M1. FISH was imaged with a Zeiss 510 Meta confocal microscope.

### Quantitative real-time PCR (qPCR)

Total RNA was isolated from cultures of various embryonic stages. The RNA was extracted with Eurozol (EuroClone, Celbio, Milan, Italy). The samples were treated with DNase I (Ambion, Life Technologies, Carlsberg, CA, USA) to remove DNA contamination as described by the manufacturer. First-strand cDNA was synthesized in a 20-μl reaction from 1 μg of total RNA using the SprintTM RT Complete-Double PrePrimed kit (Clontech, Saint-Germain-en-Laye, France ) according to the manufacturer’s protocol. The cDNA obtained was directly used for further studies. Specific primer sets for *Sum1/MyoD1, MyoD2, MyoR2, Twist, Maf, Myocardin, MHC, Tropomyosin1* and *Tropomyosin2* (see Additional file 1: Table S1) were designed using the Primer3 program [47] (http://bioinfo.ut.ee/primer3-0.4.0/primer3/). Primer efficiencies exceeded 1.9. Primer sets were chosen to amplify products 100 to 200 bp in length. cDNA was diluted to a nominal concentration of 1 embryo/μl. The qPCR was conducted as described [48] using the ViiA 7 REAL TIME PCR detection system and SYBR green chemistry (Applied Biosystems, Foster City, CA, USA). For all qPCR experiments, the data from each cDNA sample were normalized against the ubiquitin mRNA, which remains relatively constant during development [39,49,50]. For absolute quantification of the number of transcripts, *Z12-1* was used as an internal standard for each cDNA preparation. The number of *Z12-1* transcripts in embryos of the relevant stages had been measured earlier by RNA titration [51].

## Results and discussion

### Myoblast progression during sea urchin development: a molecular view

**Figure 1:**
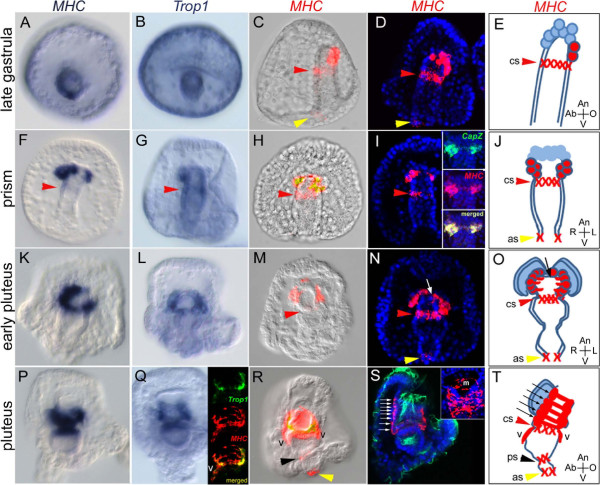
**Progression of *MHC*-positive cell lineages during sea urchin development.***MHC* and *Trop1* expression in the gastrula (40 to 48 h), prism, (48 to 60 h), early (60 to 72 h) and late pluteus stage (72 to 96 h) . Expression of *MHC***(A, F, K, P) **and *Trop1***(B, G, L, Q)** was localized by colorimetric *in situ* hybridization. The embryos in panels **A and B** are shown in a vegetal view; the ones in panels **C**-**T** are viewed along the animal top/vegetal down (A/V) axis. The inset in panel **I** is double fluorescent *in situ* hybridization (FISH) showing the co-expression of *CapZ* (green) and *MHC* (red) in myoblasts. Double FISH indicates that *Trop1* (green) and *MHC* (red) are co-expressed in the muscles of a late pluteus (inset in **Q**). FISH was used to localize the expression of *MHC* (red; **C, D, H, I, M, N, R, S)**. Bright field images **(C, H, M, R)** and confocal stacks **(D, I, N, S)** that include 4',6-diamidino-2-phenylindole (DAPI) (blue) staining are shown. Embryos in **C, D, E** and **T** are shown as lateral views with the oral side to the right. The ciliary band and gut internal cilia were stained anti-acetylated tubulin (shown as green in **S**). The inset is a magnified view of the muscle fibers from a single confocal plane. A schematic representation of the progression of MHC-positive cells in the formation of the muscle fibers is shown **(E, J, O, T)**. Ventrolateral processes (v), the cardiac sphincter (cs; red arrow), the pyloric sphincter (ps; black arrow) and the anal sphincter (as; yellow arrow) are indicated. Muscle fibers are indicated as white **(N, S)** or black **(O, T)** arrows.

To follow the progression of myoblasts during sea urchin development and describe the myogenic process from the earliest stages, we analyzed the expression of evolutionarily conserved terminal differentiation genes of the muscle gene battery such as MHC class II and Tropomyosin homologues. The myosin gene family has been preliminarily characterized in other sea urchin species [52]. In *Lytechinus variegatus,* WMISH and immunohistochemistry indicate that a Myosin heavy chain is specifically expressed in muscles at the pluteus stage [28]. This protein was also detected in *S. purpuratus* and *Lytechinus pictus* using an antibody generated against the *L. variegatus* protein [53]. An MHC class II homologue, *MHC18,* and two Tropomyosin genes, *Tropomyosin1* and *Tropomyosin2,* were cloned and characterized (see Additional file 3: Figure S1 and Additional file 4: Figure S2, respectively) for their temporal and spatial expression (see Figure 1 and Additional file 5: Figure S3). Moreover, another muscle terminal differentiation gene coding for an F-acting capping protein beta subunit (*CapZ*) was cloned and characterized by WMISH (see insert in Figure 1I) [54].

*MHC18* is orthologous to the previously described *L. variegatus* sequence (see Additional file 3: Figure S1) and is herein called *MHC*. In *S. purpuratus*, *MHC* is not expressed before the late gastrula stage (48 h), when transcripts are first detectable in one or two individual mesenchymal cells at the oral vegetal side of the tip of the archenteron (Figure 1C-E). In some cases, the expression of *MHC* appears simultaneously in each of two bilaterally symmetrical cells at the tip of the primitive gut. At the same time, *MHC* expression occurs in a few endodermal cells at the future cardiac sphincter (see red arrowheads in Figure 1) and to a lesser extent, at the anal sphincter (see yellow arrowheads in Figure 1). As the embryo progresses from the late gastrula to pluteus stage, an increasing number of neighboring cells express *MHC*. It is unclear whether this is due to proliferation or/and to independent myoblast specification. At the prism stage (55 to 60 h), the paired coelomic pouches start to extend laterally and *MHC* transcripts become localized in two rows of myoblast cells (as shown in Figure 1J). At the early pluteus stage (65 to 72 h), the two coelomic pouches become physically separated and processes of the myoblasts from each pouch extend toward the midline of the esophagus (see arrows in Figure 1N and O). In each coelomic pouch, 14 to 17 mesenchymal cells are present, of which 7 to 8 cells express *MHC*. Finally, at the late pluteus stage (72 to 96 h), myoblast processes fuse to form muscle bands (see arrows in Figure 1S and T) and ventrolateral processes expressing *MHC* extend from the ends of the coelomic epithelium. Finally, *MHC* expression is observed at the pyloric sphincter (see black arrowheads in Figure 1R and T). Double FISH experiments revealed co-expression of *Tropomyosin1* and *MHC* as well as *CapZ* and *MHC* in the circumesophageal muscles at the prism and pluteus stage (see insert in Figure 1I and Q). The findings of this molecular study parallel the morphological observations made by Burke and Alvarez (1988). Furthermore, they demonstrate that the first cells expressing markers of muscle cell differentiation are present as early as the late gastrula stage, which is well before morphological changes are evident.

### Identification and characterization of putative sea urchin muscle regulators

**Figure 2:**
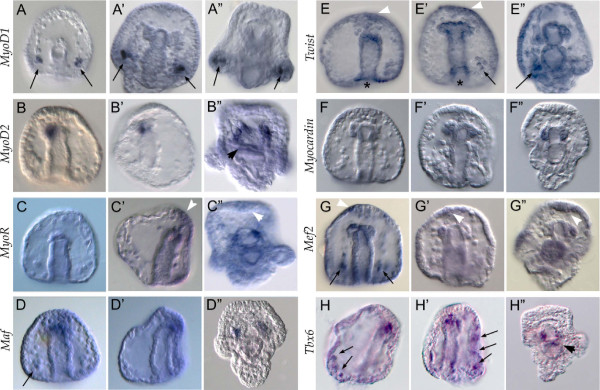
**Whole mount *in situ *hybridization (WMISH) of sea urchin regulatory genes whose orthologs are known myogenic factors.***Sum1/MyoD1, MyoD2, MyoR, Maf, Myocardin, Mef2, Twist* and *Tbx6* expression was localized using WMISH at the gastrula (44 to 48 h; **A**-**H**), prism (60 to 65 h; **A’**-**H’**), and pluteus larva stage (72 to 80 h; **A”-H”**). All embryos are viewed along the animal top/vegetal down axis with the exceptions of panel **A**, which is shown in a vegetal view with the oral side on the bottom, **B’, C’, D’** and **H** are viewed in a lateral view with the oral side on the left **(B’)** or right **(C’, D’ and H’)**. Domains of expression other than the tip of the archenteron and the coelomic pouches are indicated as follows: black arrow, primary mesenchyme cells (PMC); black arrowhead, the cardiac sphincter; white arrowhead, the apical organ; black asterisk, the blastopore.

**Figure 3:**
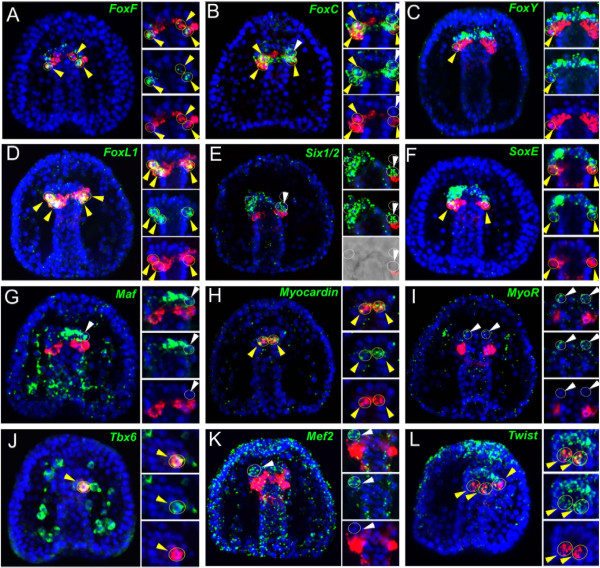
**Co-expression analysis of putative sea urchin myogenic factors and *MHC *by double confocal fluoresecent *in situ *hybridization (FISH).** Relative spatial domain of expression of *FoxF***(A)***, FoxC***(B)***, FoxY***(C)***, FoxL1***(D)***, Six1/2***(E)***, SoxE***(F)***, MaF***(G)***, Myocardin***(H)***, MyoR2***(I)***, Tbx6***(J)***, Mef2***(K)***,* and *Twist***(L)** (green) with respect to *MHC* (red) by double FISH in the late gastrula stage, 48 to 50 h. Every picture is a full projection of merged confocal stacks. Aside each picture are separately placed single focal planes of each channel plus a merged picture of an enlarged detail at the tip of the archenteron, to clarify any misleading issue of co-expression domains. In **E**, a single brightfield slice is also superimposed to the red channel. Yellow circles indicated by yellow arrowheads show co-expressing cells, and the white ones point to absence of co-expression. The white arrows indicate other domains of expression. All the embryos are viewed along the animal top/vegetal down (A/V) axis from the oral or aboral surface, excluding the one reported in **L** which is shown in a lateral view along the A/V axis with the oral side on the right. Nuclei are stained blue with 4',6-diamidino-2-phenylindole (DAPI).

To identify potential regulatory factors involved in sea urchin myogenesis, a candidate gene approach was applied. Two different gene sets were analyzed: 1) sea urchin orthologs of transcription factors, for which a well-known myogenic role has been described in one or more model species, and 2) sea urchin mesodermal transcription factors that are known to be expressed at the tip of the archenteron, which is the site of myogenesis initiation. For each of these candidate genes, expressed sequences were isolated and temporal and spatial expression were analyzed throughout sea urchin embryogenesis (see Additional file 6: Figure S4, Additional file 7: Figure S5 and Additional file 8: Figure S6). Fifteen transcription factor genes were further characterized in two steps. First, gene expression was localized using WMISH to identify genes that were expressed in the archenteron tip. Following this preliminary screen, co-expression experiments with the muscle differentiation marker *MHC* were performed for each of the putative myogenic regulators at the late gastrula stage to identify transcription factors with a potential role in controlling sea urchin myogenesis. The results of this two-step analysis are shown in Figure 2 and Figure 3, respectively.

In a closely related sea urchin species, *L. variegatus*, the gene *Sum1* (sea urchin myogenic factor 1) has been described as an MRF, although it does not appear to be a clear ortholog of any specific vertebrate myogenic bHLH factor (see Additional file 6: Figure S4). The *L. variegatus* Sum1 protein is found at the tip of the archenteron, which coincides with the location of the muscle precursors [7,37], and the mRNA is also present in ventrolateral clusters of five to six cells on either side of the archenteron. These cells are known to be the skeletogenic mesoderm, which is not known to be myogenic [37]. In *S. purpuratus*, *Sum1* mRNA appears to be expressed in an identical pattern as in *L. variegatus*[37]*.* Analysis of the *S. purpuratus* genome reveals three *MyoD* paralogues: the previously identified *Sum1*, which was annotated as *MyoD* and is herein called *MyoD1*, as well as *MyoD2* and *MyoD3*. The qPCR analysis indicates that the temporal expression profiles of *MyoD1* and *MyoD2* are unique (see Additional file 7: Figure S5). *MyoD1* begins to be significantly expressed at 30 to 36 h, the time period during which skeletogenesis occurs. In contrast, *MyoD2* zygotic expression is detected starting from 45 to 48 h, which is concurrent with the appearance of the first cells expressing myogenic differentiation genes (see above), and expression continues through the pluteus larva stage. Phylogenetic analysis suggests that *MyoD2* is more closely related to the *Drosophila* ortholog *nautilus* than *Sum1/MyoD1* (see Additional file 6: Figure S4). WMISH using a gene-specific probe that lacks the highly conserved bHLH domain shows a different expression pattern for *Sum1/MyoD1* than that already described [37]. We found that *MyoD1* is expressed exclusively in the skeletogenic mesoderm in all developmental stages examined. Transcripts of this gene were never found at the tip of the archenteron where myogenesis initiates (Figure 2 A-A”). Together, these findings suggest that the function of *MyoD1* is restricted to regulating skeletogenesis. Interestingly, *MyoD2* expression appears to be specific to the myogenic region. At the late gastrula stage (45 to 48 h) *MyoD2* is weakly expressed in a few cells at the oral side of the tip of the archenteron. Later, in the prism (60 to 65 h) and pluteus larva (72 to 80 h) stages, transcripts of this gene are found in the coelomic pouches (Figure 2 B-B”).

*MyoR, Twist, Tbx6, Mef2, Myocardin* and *Maf* genes were analyzed either for their known myogenic role or for their involvement in the differentiation and development of a variety of tissues, as in the case of *Maf*[55]. These genes exhibit a common expression pattern. During gastrulation, they are expressed at the tip of the primitive gut, whereas later in development they are localized in the coelomic pouches (see Figure 2). Other expression domains include scattered cells of the NSM (*MyoR*: Figure 2C and Figure 3I), the primary mesenchyme cells (PMCs), which form the larval skeleton (*Maf, Twist, Tbx6* and *Mef2*: Figure 2D, E-E”, H, H’ and G; see also Figure 3G and J and Additional file 9: Figure S7), and the blastopore and apical ectoderm (*MyoR, Twist* and *Mef2*: Figure 2C’-C”, E-E’ and G-G”). Moreover, *MyoD2* and *Tbx6* are also seen in the presumptive cardiac sphincter (Figure 2B” and E”). *FoxC, FoxF, FoxL1* and *FoxY*[39,40,56,57], *SoxC* and *SoxE*[35,42] and *Six1/2* and *Eya* genes [56,58] were included in this study due to their described expression at the tip of the archenteron, which was recapitulated here (see Additional file 8: Figure S6).

Finally, to ascertain the molecular identity of the sea urchin myoblasts, each of the selected candidate genes was tested separately for expression in myoblasts at the onset of myogenesis (48 to 50 h) by double FISH analysis, using *MHC* as a myoblast marker. From all of these transcriptional regulators, only *FoxF, FoxC, FoxL1, Myocardin, Twist* and *Tbx6* overlap with *MHC* in the hereby defined myogenic domain, that is, the most vegetal portion of mesenchymal cells, which emerge from the oral side of the archenteron tip. This expression domain corresponds with the morphological evidence and makes them strong candidates for myogenic factors (Figure 3). *MyoD2* expression is also restricted to the myogenic domain (Figure 2B-B”). *FoxY* and *SoxE* are co-expressed with *MHC* only in a few cells at the border between the myogenic oral domain and the aboral side of the tip of the archenteron (Figure 3C and F). None of the other transcription factors analyzed showed significant co-localization at the myogenic domain (see Figure 3 and Additional file 9: Figure S7). The high resolution of the confocal analysis allowed us to observe that the co-expression of *MHC* and these putative myoblast regulators always and exclusively occurs in cells of typical mesenchymal shape that are clearly distinct from the endodermal epithelium at the tip of the archenteron to which they remain attached (for example, see detail in Figure 3E). This implies that at the onset of *MHC* expression, these mesodermal cells have already undergone the epithelial-mesenchymal transition.

### The mesoderm at the tip of the late gastrula archenteron is subdivided in distinct regulatory states

**Figure 4:**
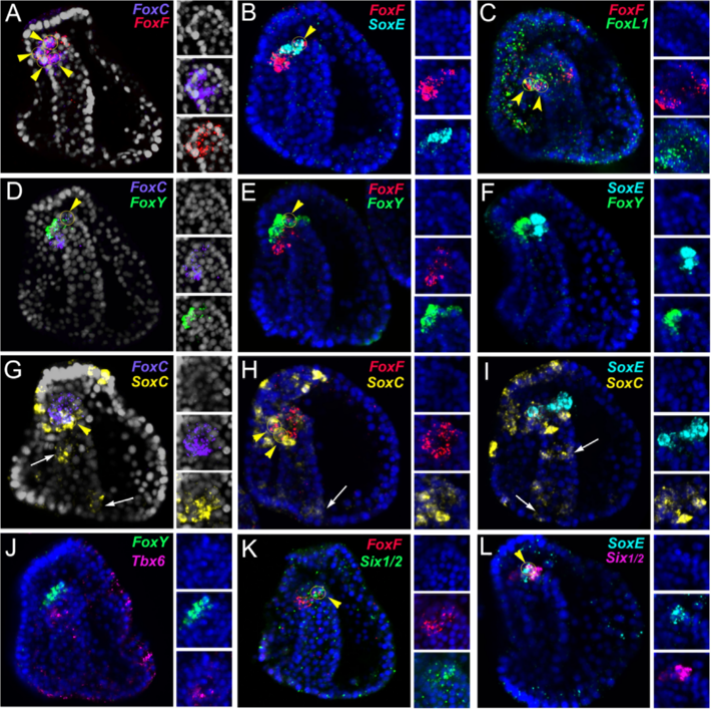
**Co-expression analysis of sea urchin putative regulatory factors at the tip of the archenteron by double confocal fluorescent *in situ *hybridization (FISH).** Relative disposition of *FoxF***(A-C, E, H and K)***, FoxC***(A, D and G)***, FoxY***(D-F and J)***, FoxL1***(C)***, Six1/2***(K and L)***, SoxE***(B, F, I and L)***, Tbx6***(J)** and *SoxC***(G-I)** transcripts by double FISH in the late gastrula stage, 48 to 50 h. Every picture is a full projection of merged confocal stacks. *FoxF* is stained in red, *FoxY* and *FoxL1* in green, *FoxC* in purple, *Six1/2* and *Tbx6* in magenta, *SoxE* in cyan and *SoxC* in yellow. Panel **K** is the only exception where *Six1/2* is depicted in green. Full projection of split channels showing enlarged details of the tip of the archenteron are placed aside each picture. Yellow circles indicated by yellow arrowheads show cells co-expressing the analyzed genes. White arrows in **G**-**I** indicate the position of the presumptive anal and cardiac sphincters. The ectodermal staining in **C** corresponds to ciliary band expression of the *FoxL1* gene. All the embryos are viewed in a lateral view along the animal top/vegetal down axis with the oral side on the left. Nuclei are labeled blue with 4',6-diamidino-2-phenylindole (DAPI).

**Figure 5:**
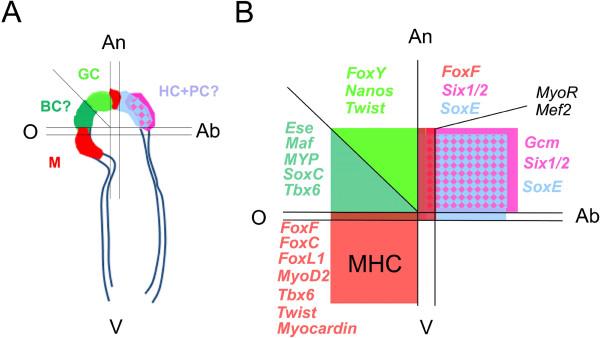
**Map of the regulatory state of the main mesodermal domains of the tip of the sea urchin archenteron at the late gastrula stage. (A)** Schematic representation of a 48-h sea urchin embryo archenteron in lateral view along the animal top/vegetal down axis. The cell fate of each region is indicated as follows: Ab, aboral; An, animal; BC, blastocoelar cells; GC, germ cells; HC, hydropore canal; M, muscles; O, oral; V, vegetal. **(B)** Different mesodermal regions identified by specific regulatory signatures at the tip of the archenteron are shown in different colors distributed over the three major domains defined in this study. Regions of partial overlaps and names of the genes expressed in each region are shown in colors associated with each domain.

The data described above identified seven relevant transcription factors, each expressed in a subset of *MHC*-positive cells. To obtain a high-resolution map of the molecular identity of the myogenic domain and the neighboring mesodermal domains located at the tip of the fully invaginated archenteron, double FISH experiments were performed to identify different combinations of factors that are co-localized in the domain of interest. The results of this analysis are reported in Figure 4 (summarized in Figure 5) and reveal a complex subdivision of the mesodermal cells at the tip of the archenteron. To better describe the topology of the different domains at the tip of the archenteron, and given the fact that in *S. purpuratus* the archenteron is curved, we orientated these domains along the oral/aboral and animal/vegetal axis: aboral animal (AbAn), for the mesodermal domain at the tip of the archenteron that faces the aboral ectoderm; oral animal (OAn), describes the mesodermal domain at the tip of the archenteron that faces the oral ectoderm and is closer to the animal pole; and oral vegetal (OV), which is the mesodermal domain at the tip of the archenteron that faces the oral ectoderm and is closer to the vegetal pole, defined also as the myogenic region due to the early appearance of the *MHC* gene in this domain. *FoxC, FoxL1* and *FoxF* are co-expressed in a significant group of cells within the myogenic domain (see Figure 4A, C and compare with Figure 1D and Figure 3A, B). However, *FoxF* also shows an additional small domain of expression at the aboral side of the tip of the gut (Figure 4A), where it is co-expressed with *Six1/2* (Figure 4K) and *SoxE* (Figure 4B). This is consistent with the co-localization of *Six1/2* and *SoxE* in the same aboral cells (Figure 4L), while also presenting a broader domain of expression. Moreover, both *FoxC* and *FoxF* are co-expressed with *FoxY* in a few cells at the periphery of the myogenic domain (Figure 4D and E). This is in agreement with the partial overlap of *FoxY* with *MHC* at the late gastrula stage (Figure 3C). *Maf* and *SoxC* are also seen in some mesenchymal cells at the border between the myogenic domain and the small micromere (SM) derivatives, together with *Ese*[41] and *Vitellogenin/Major yolk protein* (*MYP*) (Figure 3G and 4G and H; see also Additional file 9: Figure S7). *SoxC* expression, however, is not restricted to these cells. It is also present in scattered endodermal cells, in particular the region just below the myogenic domain (Figure 4H, I), which coincides with *Brn1/2/4* expression [59] (Additional file 9: Figure S7). Additionally, *SoxC* expression is observed in the presumptive gut sphincters and in several scattered ectodermal cells of the ciliary band and apical organ (Figure 4G-I).

From this large-screen approach, we are able to generate a model that simplifies the compartmentalization of mesoderm and provides a regulatory-state map of the mesoderm at the tip of archenteron at the late gastrula stage (48 h) (Figure 5). The mesoderm archenteron tip can be divided into three distinct domains: the OAn, the OV and the AbAn domains. Each domain has a different molecular identity, and therefore a specific regulatory state, defined as the cohort of transcription factors and signaling molecules that are co-expressed in it. The OV domain corresponds to the myogenic region of the embryo and expresses the regulatory genes *FoxC, FoxF, FoxL1, Myocardin, MyoD2, Tbx6* and *Twist,* as well as the terminal differentiation genes *MHC* and *Tropomyosin1.* The OAn domain can be subdivided into two sub-domains: a larger one that is adjacent to the myogenic domain, where *Ese, Tbx6, Maf, SoxC* and *MYP* are expressed; and a smaller one that derives from SM descendants, points towards the animal pole of the embryo and expresses only *FoxY, Nanos* and *Twist*. *FoxY* is known to be expressed at the very tip of the archenteron together with the germ cell markers *Nanos* and *Vasa*[57,60]. These cells contribute to the primordial germ cell lineage of the adult [42]. Finally, the AbAn domain expresses *FoxF, Gcm, MyoR, Mef2, Six1/2* and *SoxE* with some of the genes occupying smaller or larger domains of expression as described before and reported in detail in Figure 5 (see also Additional file 9: Figure S7).

The cell fate of each of these domains in the late larval stages has not yet been fully determined. What is known so far is that the smaller OAn domain that expresses *FoxY, Twist* and *Nanos* genes will contribute to the formation of the adult rudiment and its integrity is essential to the reproductive potential of the adult [57,60]. Also, some of the genes found in the AbAn domain, such as *Six1/2, Eya* and *SoxE*, were recently demonstrated to be involved in specifying the hydropore canal originating from the left coelomic pouch of the pluteus larva [56]. Although *Gcm* and *Six1/2* are known to be involved in pigment cell formation [61], these cells have probably already migrated to the ectoderm at this stage, leaving the cell fate of the AbAn domain in the right coelomic pouch still an open question. Similarly, while it is known that presumptive blastocoelar cells express *Ese*[41,62,63] and most of them delaminate from the tip of the archenteron at the late gastrula stage, it remains unclear whether a subset of these cells are still present in the OAn domain of the tip of the archenteron at the end of gastrulation. Finally, as suggested in this study, the OV domain will give rise to the esophageal muscle fibers (Figure 5A).

### Myoblast precursors are identified by a specific regulatory state at the beginning of gastrulation

This analysis identified the molecular fingerprint of myoblasts at the onset of myogenesis in the sea urchin embryo (48 h) without revealing where these cells come from and when they are specified as myoblast precursors. To better understand the origin and the molecular identity of the sea urchin myoblast precursor cells, we looked at gene expression in the very early gastrula stage (30 h), when the different NSM lineages are already segregated. Two members of the Fox family (*FoxC* and *FoxF*) that showed a significant overlap of expression with *MHC* at the onset of myogenesis (48 h) were chosen as putative markers of the myoblast precursors due to their early onset of expression in the NSM during gastrulation [40]. The detailed temporal expression profiles available by nanostring data [50] (see Additional file 10: Figure S8) were integrated with cellular resolution analysis of the spatial expression of the selected genes at early developmental stages. *FoxY* was also included in this analysis, given its partial co-expression with *MHC* at late gastrula stage (Figure 3C) as well as its previously reported expression in a subset of NSM at early gastrulation [39]. Finally, during the preparation of this manuscript, a paper was published suggesting a functional role of *FoxY* in sea urchin muscle formation [31].

**Figure 6:**
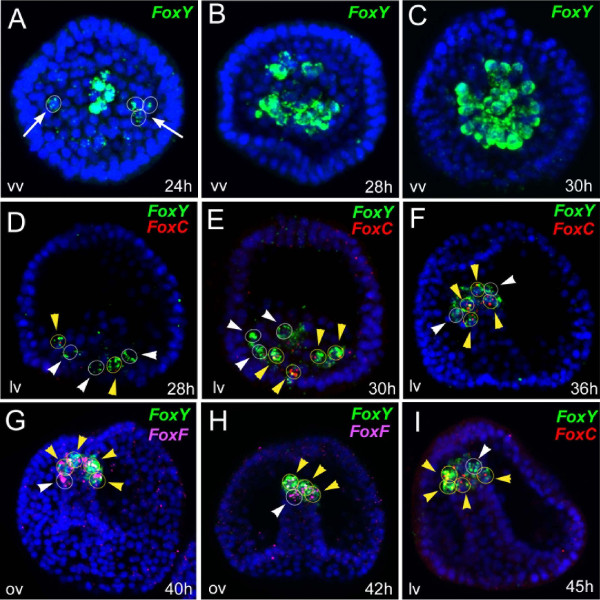
**Dynamics of gene expression in the putative myoblast precursors of the sea urchin embryo.** Fluorescent whole mount *in situ* hybridization and relative position of *FoxY* (green), *FoxC* (red) and *FoxF* (magenta) transcripts in the interval from 24 to 45 h. Each picture is a full projection of merged confocal stacks. Yellow circles indicated by yellow arrowheads show co-expression cells; white circles show absence of co-expression. White arrows in panel **A** indicate the emergence of *FoxY* transcription in the NSMs. For single-channel full projections of the images reported in **D-I** see Additional file 11: Figure S9. All the embryos are viewed in lateral view along the animal top/vegetal down axis with the exception of **A, B and C** that are seen in a vegetal view. Nuclei are labeled blue with 4',6-diamidino-2-phenylindole (DAPI).

A striking observation arises from the temporal expression profile of *FoxY, FoxC* and *FoxF* during sea urchin gastrulation (Figure 6, see also Additional file 11: Figure S9). In particular, the emergence of *FoxC* expression corresponds to the enrichment in the number of *FoxY* transcripts (see nanostring expression profiles in Additional file 10: Figure S8). Using a detailed confocal analysis in a series of developmental stages, starting from the time of the first appearance of *FoxY* transcripts, we characterized the dynamic expression pattern of this gene in both SM descendants and part of the NSM (Figure 6A-C and Additional file 10: Figure S8). The emergence of *FoxY* expression in the NSM likely coincides with the only duplication event that occurs in the SM derivatives (24 to 28 h), resulting in a total of eight cells (Figure 6B and C). Indeed, at 30 h, although an average of 20 *FoxY* expressing cells is observed, only four NSM cells co-express *FoxC* (Figure 6D, E and Additional file 10: Figure S8). A few hours later, at the mid gastrula stage (36 to 40 h), these cells continue to co-express *FoxC* and *FoxY,* with *FoxC* progressively in more cells, and *FoxY* in fewer cells (Figure 6F, I and Additional file 10: Figure S8). *FoxF* expression starts to be significant at the mid gastrula stage (40 h) and is only seen in a specific subset of cells that also express *FoxY* (Figure 6G and H). *FoxY* transient expression in a large NSM population is observed until the late gastrula stage (48 h), when *FoxY* transcripts are no longer detectable in *FoxF/FoxC* expressing cells (Figure 4A, D, E and Additional file 10: Figure S8) and remains restricted to the SM descendents. Remarkably, the *FoxF/FoxC* expressing cells at this stage also start to express the *MHC* gene, thus establishing the myogenic lineage (Figure 3A and B).

**Figure 7:**
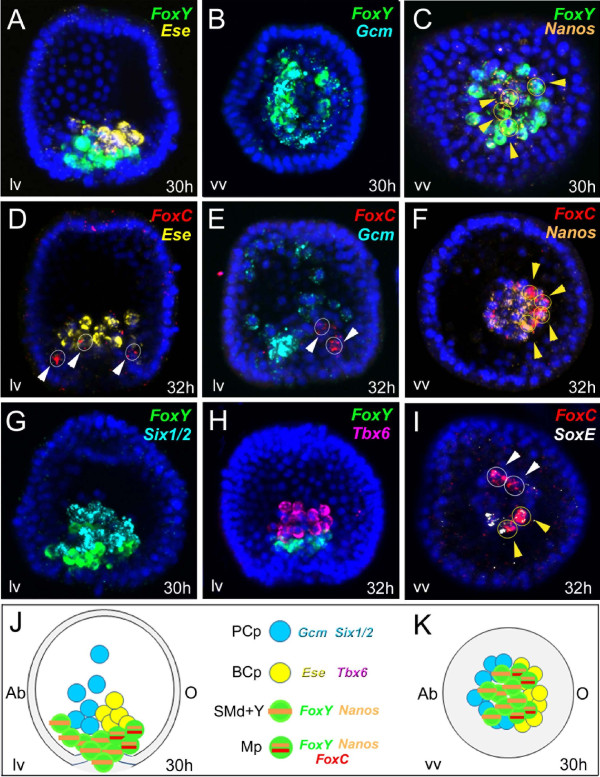
**Co-expression analysis of sea urchin putative myogenic factors and non-skeletogenic mesoderm (NSM) and small micromere (SM) molecular markers. (A**-**I)** Expression of *FoxC, FoxY, Ese, Gcm, Six1/2, SoxE, Tbx6* and *Nanos* was localized by double confocal fluorescent *in situ* hybridization at the very early gastrula stage, 30 to 32 h. Every picture is a full projection of merged confocal stacks. Yellow circles indicated by yellow arrowheads show co-expressing cells, and the white ones show absence of co-expression. For single-channel projections of the images reported in **C, F and I** see Additional file 11: Figure S9. All the embryos are viewed in a vegetal view with the exception of **A, D, E, G and H** that are seen in lateral view along the animal top/vegetal down axis. Nuclei are labeled blue with 4',6-diamidino-2-phenylindole (DAPI). In panels **J and K** a schematic representation is shown of a regulatory stage map of NSM of a 30-h sea urchin embryo orientated along the oral right/aboral left (O/Ab) axis in both lateral **(J)** and vegetal views **(K)**. The different mesodermal cell types identified by specific regulatory signatures at the vegetal plate are shown in different colors: Mp, putative myoblast precursors, green with orange and red horizontal lines; BCp, blastocoelar cell precursors, yellow; PCp, pigment cell precursors, blue; SMd + Y, small micromere derivatives (SMd) plus *FoxY* expressing NSM cells (Y), green with an orange horizontal line. A legend indicates the names of the genes expressed in each region. For the sake of simplicity, primary mesenchyme cells are not shown.

Once the putative myoblast precursors were identified, to distinguish the regulatory states of the different NSM precursors located in the vegetal plate of the early gastrula embryo, we used known molecular markers of two other well-characterized NSM lineages, the pigment cell marker *Gcm*[39] and the blastocoelar cell lineage marker *Ese*[41]. Specifically, *Gcm* is first expressed in a ring of cells that corresponds to the entire NSM lineage at the end of the cleavage stage and is under the direct control of Delta/Notch signaling [39]. By the blastula stage, *Gcm,* which is a key driver of the pigment cell regulatory program, becomes restricted to the aboral quadrant of the NSM. Simultaneously, the cells located in the oral region of the NSM, which later give rise to the blastocoelar cells, start to express a blastocoelar cell regulatory program that includes genes such as *Ese, GataC* and *Scl*[62,64]. Finally, we used *Nanos* expression as a means to distinguish the SM lineage [42]. Using FISH we have identified four cells that co-express *FoxY* and *FoxC* located on the oral side of the vegetal plate (facing the future oral ectoderm) at the very early gastrula stage (28 to 32 h). These cells never express other NSM markers such as *Gcm* (Figure 7B and E)*, Ese* (Figure 7A and D) or *Six1/2* (Figure 7G). However, they do transiently express the germ cell marker *Nanos* (Figure 7C and F) and, in part, *SoxE* (Figure 7I)*.* Moreover, they never express *Tbx6,* which at this developmental stage is only seen in the blastocoelar cell precursors (Figure 7H; see also Additional file 9: Figure S7).

The high-resolution spatial-temporal analysis coupled with co-localization data enables the description of the molecular signature of the putative myoblast precursors at their early onset. Furthermore, integration of all the data provides a cellular resolution map of the NSM at the very early gastrula stage (30 h) (Figure 7J and K), in which the precursors of different cell types are only identified by a unique molecular identity. When viewed from the vegetal plate (Figure 7K), the ring of NSM can be divided in different cell populations as follows. The four putative myoblast precursors are found in the oral/lateral side of the vegetal plate, residing along the animal-vegetal axis, between the blastocoelar cell precursors and the vegetal pole, where SM descendants are located and express *FoxY, FoxC*, *Nanos* and, partially, *SoxE*. Pigment cell precursors, expressing *Gcm* and *Six1/2,* are excluded from the oral region and appear more apical with respect to the invaginating archenteron, while blastocoelar cell precursors, expressing *Ese* and *Tbx6*, are excluded from the aboral region. Finally, the eight SM descendants are established mostly in the central part of the vegetal pole, more vegetal with respect to pigment and blastocoelar cell precursors.

In summary, the esophageal muscles in sea urchin larva appear to be specified in the following order: at the very early gastrula stage (30 h), four NSM cells are committed to adapt the myogenic fate. Following a likely signaling event, these cells start to express *FoxY, FoxC* and partially *SoxE* as part of the muscle specification gene battery. At the mid gastrula stage (40 h), these putative myoblast precursors, which are positioned at the oral side of the tip of the primitive gut, start to express *FoxF* and, later, *FoxL1.* Morphogenetic movements then occur, which results in a migration of the presumptive muscle cells along the inner side of the coelomic pouches. At the late gastrula stage (48 h), the muscle differentiation gene battery is expressed, including *MyoD2* and *Tbx6*. The emergence of myoblasts occurs at the oral vegetal domain of the tip of the archenteron by expressing the muscle terminal differentiation genes *MHC* and *Tropomyosin1*. Finally, at the prism stage, an arrangement of myoblasts in two rows surrounding the foregut takes place. These cells dissociate from the coelomic epithelium, extend processes and, finally, fuse to form muscle fibers that run parallel to each other in the pluteus larva.

From these data, we are able to trace the putative esophagael myogenic precursors back to early stages of gastrulation. This clearly supports a segregation of the myogenic lineage from the rest of the NSM as early as the very early gastrula stage. However, experiments at earlier stages of development, using a combination of NSM and endodermal markers are needed to clarify whether these putative myogenic precursors are naïve cells destined to adapt the myogenic fate or are part of any of the other known NSM subpopulations that reacquire their developmental potency and become re-specified as muscle progenitors. In either case, these cells display a certain developmental multipotency as they transiently co-express a germ cell marker, *Nanos*, which is known to be transcribed not only in germ cell lineages but also in pluripotential stem cells [65,66]. Moreover, the expression of *FoxC, FoxY,* and *SoxE* in the putative myogenic precursors (30 h) indicates that in very early gastrula stage, the NSM lineages transiently express the same transcriptional regulators, whereas later in development (48 h) these lineages acquire different molecular signatures, probably through a regulatory-state exclusion mechanism [67,68]. Perturbation analyses of the putative myoblast regulators identified in this study need to be performed to reveal the regulatory mechanisms that underlie myogenesis. However, we can already speculate that the formation of the diverse muscle cell types of the sea urchin embryo, which arise from different embryonic layers, mesoderm and endoderm, appears to be controlled by different and independent regulatory mechanisms, since none of the regulatory factors identified in this study which are specifically expressed in esophageal myoblasts are ever expressed in endodermal sphincter muscle cells during embryonic development. Unpublished data on the control of *MHC* expression in pyloric sphincter muscles also support this conclusion (Arnone, unpublished).

### The repeated use of the same genetic regulatory apparatus: conservation and divergence

Muscle development has been used as a paradigm of evolutionary conservation of cell type specification and differentiation. Even if many genes involved in muscle formation have been conserved during bilaterian evolution, such as MRFs and differentiation genes, our data show that many evolutionary differences are also present. In vertebrates, the bHLH-containing MRF group including MyoD and the other MRFs in vertebrates, was derived by gene duplications from a single ancestral *MyoD* gene [69]. This is proven by the fact that in invertebrates only a single member of the *MyoD/MRF* gene family exists and its role is evolutionarily conserved during myogenesis. In the majority of the cases found in the literature, *MyoD* or other equally related MRFs, for example, the ascidian *Ci-MRF* gene [70], have important roles during myoblast specification. In the sea urchin, out of the three paralogs found in the genome, only one, *MyoD2*, has an expression pattern consistent with a role as an MRF, whereas *Sum1/MyoD1* appears to have been co-opted to serve the skeletogenic lineage. The expression profile of *MyoD2*, together with the phylogenetic analysis (see Additional file 6: Figure S4), strongly suggest *MyoD2* involvement in sea urchin embryonic myogenesis and explain the already published expression profile of *MyoD1* at the tip of the archenteron as a cross-reactivity case of the mRNA riboprobe used in the WMISH experiments. In fact, the two genes are identical at 66% of their nucleotide sequence in the bHLH domain. Therefore, *MyoD1* stands as an example of neofunctionalization, a principle that contributes to retention of duplicate genes by providing them new functions and generating divergence.

Two other *bHLH* factors known for their involvement in myogenesis were analyzed in this work, MyoR and Twist. MyoR is a myogenic repressor in most of the animals studied [15,16,71], and Twist acts as a repressor in vertebrates [72] and is a myogenic activator in protostomes [73]. The fact that in sea urchins, *MyoR* and *Twist* are expressed in NSM cells other than the myoblast precursors, could suggest a repressive action of these genes on the muscle gene battery and perhaps reinforce their general role as myogenic repressors. On the other hand, our co-expression analysis also revealed a partial localization of *Twist* in the myogenic domain (Figure 2). The only available data in the relevant literature on a putative functional role of *Twist* in the sea urchin is on *LvTwist*, where it has been suggested that this factor is somehow acting as a myogenic activator [30]. However, these perturbation experiments were performed in another sea urchin species (*L. variegatus*) where the pattern of expression of *LvTwist* is slightly different from that of *S. purpuratus* in this study, where novel domains of expression are observed (apical ectoderm and blastopore). It is thus as yet unclear if these functional differences are the result of a functional switch in *L. variegatus* or convergent evolution in *S. purpuratus*. Moreover, the presence of *Twist* in the SM lineage could indicate a role in the epithelial-mesenchymal transition that the SM undergo at the prism stage (50 h) in order to migrate in the coelomic pouches [74]. A similar role of *LvTwist* is reported during the PMC ingression [30]. Finally, the two vertebrate MyoR paralogs *musculin* and *capsulin*, and the *Drosophila* ortholog *HLH54F*, are expressed in migrating mesodermal populations of myoblast progenitors [16,75]. Therefore, it is possible that parts of the regulatory circuit in the control of cell migration have also been conserved in sea urchins, since transcripts of the gene are found in scattered NSM cells.

Concerning the *Forkhead* family, *FoxC, FoxL1* and *FoxF* expression patterns in sea urchin myoblasts can also be considered as elements of conservation, as these genes show conserved expression across the animal kingdom in developing endomesodermal tissues and patterning the mesoderm, including, in some cases, their involvement in muscle development [76-78]. Moreover, comparative genomics have shown that *FoxC1* and *FoxF2,* together with *FoxQ1* and *FoxL1*, are clustered in insects, lophotrochozoans, amphioxus and vertebrate genomes and that this cluster has been maintained since the period of the early bilaterians [77,79]. As in the human genome, where *FoxL1, FoxC1, FoxF2* are located within a 300 kb region on chromosome 16, we see a similarly close linkage between *FoxL1, FoxC* and *FoxF* in the *S. purpuratus* genome, such that the genes are clustered within approximately 300 kb, with 70 kb separating *FoxC* and *FoxF* genes (see Additional file 12: Figure S10). The fact that these genes are closely clustered among so many diverse animal groups could reflect their putative interaction and further suggest the level of importance of their sequential activation, such as the one found in the sea urchin where a mesodermal co-expression is observed indicating a putative inter-regulation system during muscle specification.

*FoxC* is the earliest marker we could identify exclusively in the putative myogenic precursors, followed by the expression of *FoxF* and *FoxL1. FoxF* has an extra small domain of expression in the aboral animal domain of the archenteron, where during the next step of myogenesis at the prism stage, the appearance of muscle fibers that will contribute to the musculature apparatus surrounding the esophagus is evident. Although the expression of *FoxY* was previously described as *FoxC-like*[39,40], it is instead another example of evolutionary novelty that is specific to the sea urchin genome and is one of the first transcription factors that is observed in the putative myogenic lineage. Whether the expression of the gene in NSM precursors in evolutionary time took place prior to its localization in the SM or not remains an open question. However, the invention of new key upstream regulators of myogenesis is not unprecedented. In ascidians for example, a key myogenic factor that plays an important role in the primary muscle cell lineage specification, *Macho-1*, is a maternal factor specific to that phylum [80,81]. Also, in *C. elegans*, a unique transcription factor, FOZI-1, functions in the M lineage for the proper myoblast specification of both body wall muscles (BWMs) and coelomocytes (CCs) [82].

The *T-box* family member *Tbx6,* which is required for the regulation of muscle developmental program in vertebrates [83,84] and in *Ciona*[21], also has a conserved role in the sea urchin where it functions in myoblast patterning. However, its expression in the myogenic lineage is seen only at the late gastrula stage and transcripts of the gene are never found in the putative myoblast precursors.

Another regulatory gene expressed during myogenesis is *SoxE.* Early in development it is transiently expressed in myoblast precursors and later, in the animal aboral domain that will contribute to the formation of the hydropore canal and the adult rudiment [56]. This could reflect its putative regulation in myogenesis, possibly by having a conserved role in specifying proliferating myoblasts and repressing muscle differentiation, such as is seen in vertebrates [11]. This hypothesis could be supported by the dynamic nature of its expression pattern. In fact, after myoblasts are specified, *SoxE* is turned off in the myoblast lineage and is expressed in a separate mesodermal domain (that is, coelomic pouches) suggesting that muscle differentiation is free to occur.

One more family that has been analyzed, which has members known to be evolutionarily conserved myogenic factors, is the MADS box transcription factor family that includes *Mef2* and SRFs like *Myocardin*. The gene *Mef2* characteristically exhibits several alternatively spliced isoforms that are differentially expressed in various tissues (including muscle). This gene can also be expressed in neuronal tissues and the establishment of *Mef2* in the neuronal/apical domain is evolutionarily conserved in vertebrates, *C. elegans* and sea urchins. *Mef2* expression has also been reported in endomesodermal tissues and in the sea urchin, *Mef2* transcripts are indeed present in some endomesodermal domains, respecting the conserved dual function of the gene in these two territories, but it is never found in the myogenic domain, which suggests that its myogenic function was lost in sea urchins. On the other hand, the MADS protein *Myocardin* is localized to the myogenic region indicating its putative role in myogenesis.

## Conclusions

These findings allow for the first time 1) the description of myogenesis in the sea urchin embryo combining both morphological and molecular aspects of the process; 2) the determination of the regulatory and differentiation signatures of the sea urchin myoblasts at the late gastrula stage; and 3) the identification of the molecular fingerprint and location of the putative myoblast precursors at the very early gastrula stage. Moreover, a cell-resolution map of the NSM at the very early and late gastrula stage was established based on distinct regulatory states. To summarize, evolutionary conservation and divergence in the regulatory apparatus controlling myoblast specification was found in this study. We can conclude that of the eight proposed sea urchin myogenic regulators, only one stands as an echinoid invention. On the other hand, seven out of the nine known vertebrate myogenic regulators analyzed in this study appear to participate in the sea urchin myoblast regulatory apparatus, indicating a high level of conservation of the muscle gene battery within deuterostomes.

## Abbreviations

AbAn: Aboral-animal; BLAST: Basic local alignment search tool; BLASTP: Protein BLAST; bp: Base pairs; CapZ: F-acting capping protein beta subunit; DAPI: 4',6-diamidino-2-phenylindole; DIG: Digoxigenin; DNP: Dinitrophenol; FISH: Fluorescent *in situ* hybridization; h: Hours post-fertilization; lv: Lateral view; MAF: Musculo aponeurotic factor; MOPS: 3-(N-morpholino) propanesulfonic acid; MHC: Myosin heavy chain; MRF: Myogenic regulatory factor; MYP: Major yolk protein; NCBI: National Center for Biotechnology Information; NSM: Non-skeletogenic mesoderm; OAn: Oral animal; OV: Oral vegetal, oral view; PCR: Polymerase chain reaction; PMCs: Primary mesenchyme cells; qPCR: Quantitative polymerase chain reaction; SM: Small micromere; SRF: Serum response factor; TBLASTX: Translated nucleotide BLAST; VV: Vegetal view; WMISH: Whole mount *in situ* hybridization.

## Competing interests

The authors declare that they have no competing interests.

## Authors’ contributions

CA carried out gene orthology and phylogenetic analyses, molecular cloning, *in situ* hybridizations, microscopic and confocal imaging, qPCR, data analysis and wrote the manuscript. FR and EI carried out gene orthology and phylogenetic analyses, molecular cloning, *in situ* hybridizations, microscopic imaging and qPCR. PO contributed to the design of the study and to analysis of the data. MIA conceived the study, designed the study, contributed to analysis of the data and prepared the figures. All authors read and approved the final manuscript.

## Supplementary Material

Additional file 1: Table S1 Primers used for whole mount *in situ* hybridization (WMISH) and qPCR experiments. Click here for file

Additional file 2: Table S2 Sequences used for phylogenetic analyses. Click here for file

Additional file 3: Figure S1 Phylogenetic tree of MHC and Tropomyosin sequences. Neighbor-joining trees of MHC **(A)** and Tropomyosin **(B)** proteins from cnidarians, ecdysozoans, lophotrohozoans, placozoans, hemichordates, cephalochordates, urochordates, vertebrates and the sea urchin *S. purpuratus*. In panel **A**, *Sp-My18A* and *Sp-MyHCb* are the same protein, annotated twice. Similarly, Sp-006850 and Sp-021621 are different domains of the same protein. The trees were generated from the alignment of the amino acid sequences of the MHC proteins using CLUSTAL X and TreeView. Numbers indicate bootstrap support for given nodes. Maximum parsimony methods also confirmed all group nodes. All named proteins are appended with the species designation (one letter for the genus, one for the species). Accession numbers and sequences used are provided in Additional file 2: Table S2. Click here for file

Additional file 4: Figure S2 *MHC, Tropomyosin1* and *Tropomyosin2* temporal expression profile during sea urchin embryogenesis. Graphs show the temporal expression profile revealed by qPCR and expressed in number of molecules per embryo. Average calculations over the various measurements ± standard deviations per individual time points of development are reported as columns with error bars. Click here for file

Additional file 5: Figure S3 *Tropomyosin2* spatial expression pattern during sea urchin embryogenesis. *Tropomyosin2* whole mount *in situ* hybridization (WMISH) at the gastrula stage, 44 h **(A)** and 48 h **(B)**; prism stage, 54 h **(C)** and 64 h **(D)**; and pluteus larva stage, 68 h **(E)**, 72 h **(F)** and 84 h **(G)**. All embryos are viewed along the animal top/vegetal down axis in the oral or aboral view, with the exception of picture **G,** which is shown in a lateral view with the oral side on the left. Click here for file

Additional file 6: Figure S4 Phylogenetic tree of MRF sequences. A neighbor-joining tree was built using myogenic regulatory factor (MRF) protein sequences from humans, mouse, fish, flies and the sea urchin *S. purpuratus.* The tree was generated from the alignment of the amino acid sequences of the MRF proteins using CLUSTAL X and TreeView. Numbers give the bootstrap support for given nodes. Validation of the tree using maximum parsimony methods confirmed all group nodes. All named proteins are appended with the species designation (one letter for the genus, one for the species). Accession numbers and sequences used are provided in Additional file 2: Table S2. Click here for file

Additional file 7: Figure S5 *MyoD1/Sum1, MyoD2, MyoR2, MAF, Twist* and *Myocardin* temporal expression profile during sea urchin embryogenesis. Graphs show the temporal expression profile revealed by qPCR and expressed in number of molecules per embryo. Average calculations over the various measurements ± standard deviations per individual time points of development are reported as columns with error bars. Two *MyoR*s were identified in the sea urchin genome; *MyoR2* and *MyoR4*. From these two, only *MyoR2,* which in the current work is referred to as *MyoR*, showed significant expression as measured by qPCR. Click here for file

Additional file 8: Figure S6 Whole mount *in situ* hybridization (WMISH) of putative sea urchin myogenic regulators expressed at the tip of the sea urchin archenteron. *FoxC, FoxF, FoxY, Six1/2, Eya, SoxE and SoxC* WMISH at the gastrula stage, 44 to 48 h; prism stage, 60 to 65 h; and pluteus larva stage, 72 to 80 h. All embryos are viewed along the animal top/vegetal down axis with the exception of *FoxF-, Six1/2-* and *Eya-*stained embryo, which are shown in a vegetal view. Click here for file

Additional file 9: Figure S7 Co-expression analysis of mesodermal factors and *MHC* by double confocal fluorescent *in situ* hybridization (FISH). Relative spatial domain of expression of *Ese***(A)***, Gcm***(B)***, MYP***(C)**, *Brn1/2/4***(D)** (green) *SoxC***(E)** (green), *Nanos***(F)** (green) and with respect to *MHC* (red) by double FISH in the late gastrula (48 to 50 h) and prism stage (55 to 60 h). **(G,H)** Localization of *Tbx6* (magenta) and *Ese* (green) transcripts by double FISH in the very early gastrula (28 to 30 h) **(H)** and late gastrula stage (48 to 50 h) **(G)** are seen. Each picture is a full projection of merged confocal stacks. All embryos are viewed in an aboral or oral view along the animal top/vegetal down axis excluding the one reported in panel **A**, which is shown in a lateral view with the oral side on the left, and picture **H** that is seen in vegetal view. Nuclei are stained blue with 4',6-diamidino-2-phenylindole. Click here for file

Additional file 10: Figure S8 Relative temporal expression profiles of *FoxY, FoxC* and *FoxF* during sea urchin embryogenesis. **(A)** Temporal expression profile of *FoxY* (purple)*, FoxC* (black) and *FoxF* (yellow), as measured by nanostring data (http://sugp.caltech.edu/endomes/#HDTimecourse) [50]. Relative expression levels are shown as percent of the maximum value for each gene. Columns shown in color associated with each gene represent the number of cells per embryo expressing each gene at the given developmental times. Black lines indicate the number of SM derivatives. **(B)** Columns represent *FoxY-FoxC, FoxY-FoxC-FoxF* and *FoxC-FoxF* non-skeletogenic mesoderm co-expressing cells per embryo in colors associated to each gene from the time of their first appearance at the very early gastrula (30 h) to late gastrula stage (48 h). Click here for file

Additional file 11: Figure S9 Split-channel full projections of images **D**-**I** from Figure 6 and images **C**, **F** and **I** from Figure 7 are reported after each full merged picture. Embryo orientation and color codes are as previously reported in the legends of Figure 6 and Figure 7. Click here for file

Additional file 12: Figure S10 Genomic organization of *FoxL1*, *FoxC* and *FoxF*. An *S. purpuratus* genomic scaffold (approximately 300 Kbp) (http://www.spbase.org) is shown. This genomic region includes both predicted sequences of *FoxL1, FoxC* and *FoxF* (light blue) and transcripts (green). Click here for file
