# Supplementary material for: Myogenesis in the sea urchin embryo: the molecular fingerprint of the myoblast precursors
Source: EvoDevo. 2013 Dec 2;4:33. doi: 10.1186/2041-9139-4-33 (PMC4175510; doi:10.1186/2041-9139-4-33)
Supplement: Additional file 1: Table S1 — Primers used for whole mount in situ hybridization (WMISH) and qPCR experiments. [file 2041-9139-4-33-S1.pdf]

Table S1

Gene name	Accession number	Forward primer	Reverse primer	Use	Length of riboprobe fragment (% of cDNA covered)	Start*	End*
Sum1/MyoD1	SPU_021119	GCCGATGCAGAGGAGTAGTT	CTTGTTTCTGCCGAGTCCAT	WMISH template	552 bp (44,6%)	+5	+557
MyoD2	SPU_006232	GACAGTGCCAAGGTGGATTT	TGGGGTTAATGAGAGGGATG	WMISH template	951 bp** (93%)	-105	+846
MyoR2	SPU_012008	CAGTTCAATGAGGCCGACTT	TCTTCCATCAAGCGAGTCATC	WMISH template	477 bp (70,9%)	+45	+522
Twist	SPU_030059	TGAATTCTCTGACGGGGTCT	GTTGTCGTTCTCGCACATTG	WMISH template	609 bp (60,2%)	-248	+361
Maf	XM_003724131.1	GACGGTGATCAGACCATGTG	CTGTACTTTGCACCCGTCCT	WMISH template	2187 bp (27,8%)	+988	+3175
Eya	SPU_013869	GTATTGGAAGAGGGCGTCAA	ACTTGTTACCCGCCAGAATG	WMISH template	878 bp (83,1%)	+127	+1005
Capz	SPU_021007/ SPU_000461	AAGAAGAAGCAACGCCAAGA	GAGATCGCGACTCACAACAA	WMISH template	441 bp (29,7%)	+271	+712
MHC	SPU_010054	TGTTCCCCAAGGCTACTGAC	AAGTGGACAGCGTGCTTCTT	qPCR			
Tropomyosin1	SPU_000128	TGAATCAACAGAGCGTGAGG	TGCACCTCAGCTTCAAGAGA	qPCR			
Tropomyosin2	SPU_011034	GGGGCTGATCCAGTCAGATA	GTTGAGCGTCTTGGCTTGTT	qPCR			
Myocardin	577722	ACCAAGGTGACGAGTTCCAC	AGGAGAAGCAGGGTCAGACA	qPCR			
Ubq	SPU_021496	CACAGGCAAGACCATCACAC	GAGAGAGTGCGACCATCCTC	qPCR			
Sum1/Myod1	SPU_021119	ACGACTTCGAAAGGTGAACG	CTTGTTTCTGCCGAGTCCAT	qPCR			
MyoR2	SPU_012008	GAATGCTCGTGAACGAACTC	GCTTGAAGCAAGTCGAAGTGT	qPCR			
Twist	SPU_030059	TGAATTCTCTGACGGGGTCT	TTGGTGGTGGTAGGGTAGGA	qPCR			
Maf	XM_003724131.1	GGGTTTGCTATGGCAGTGAT	AGTGGAGAGAGGGAACAGCA	qPCR			

* Numbering refers to the translation start site (+1) based on the cDNA sequence with the exception of MyoD2 which is calculated based on the genomic sequence

** Contains also 282 bp of intron fragment
