# Supplementary material for: Myogenesis in the sea urchin embryo: the molecular fingerprint of the myoblast precursors
Source: EvoDevo. 2013 Dec 2;4:33. doi: 10.1186/2041-9139-4-33 (PMC4175510; doi:10.1186/2041-9139-4-33)
Supplement: Additional file 2: Table S2 — Sequences used for phylogenetic analyses. [file 2041-9139-4-33-S2.pdf]

Table S2

Species	Accession number/ Gene model name *	Common gene name
N. vectensis	estExt_fgenesh1_pg.C_340036 *	MyHCb
N. vectensis	estExt_fgenesh1_pg.C_1510004 *	MyHCa
C . intestinalis	NP_001071955.1	Myod1
C . intestinalis	XP_002119856.1	MyHCb
C . intestinalis	NP_001027710	Tropomyosin
D. melanogaster	AAB09048.1	Non Muscle MyHCII
D. melanogaster	NP_723999.1	MyHC
D. melanogaster	NP_476650.1	Nautilus/Myod
D. melanogaster	NP_732001	Tropomyosin1 isoform_B
D. melanogaster	NP_524321	Tropomyosin1 isoform_A
Apis mellifera	XP_393334.4	MyHC
I. scapularis	XP_002433460.1	MyHCb
P. humanus corporis	XP_002423957.1	Myosin-9
D. rerio	AAN71741.1	atrialMyHC
D. rerio	AAF00096.2	ventricolarMyHC
D. rerio	NP_001018321.1	fastMyCH4
D. rerio	NP_001018343.1	slowMyCH1
D. rerio	NP_571651.1	Myf5
D. rerio	NP_001003982	Myf6
D. rerio	NP_571337.2	Myod1
D. rerio	NP_571081	Myogenin
D. rerio	NP_957228	Tropomyosin 1 alpha
D. rerio	NP_958900	Tropomyosin 3
D. rerio	NP_001019638	Tropomyosin 4
M. musculus	Q6URW6	MyHC14
M. musculus	Q61879	MyHC10
M. musculus	O08638	MyHC11
M. musculus	P13542	MyCH8
M. musculus	NP_542766.1	MyCH7
M. musculus	Q5SX40	MyCH1
M. musculus	NP_032682.1	Myf5
M. musculus	NP_032683.1	Myf6
M. musculus	P10085	Myod1
M. musculus	NP_112466	Myogenin
M. musculus	NP_077745	Tropomyosin 1
M. musculus	NP_033442	Tropomyosin 2
M. musculus	NP_071709	Tropomyosin 3
H. sapiens	AAS98910.1	Sm MyHC1

H. sapiens	NP_005584.2	Myf5
H. sapiens	NP_002460	Myf6
H. sapiens	NP_002469.2	Myod
H. sapiens	NP_002470	Myogenin
H. sapiens	NP_998839	Tropomyosin2 isoform_2
H. sapiens	NP_001036818	Tropomyosin3 isoform_5
H. sapiens	NP_003281	Tropomyosin4 isoform_2
S. purpuratus	SPU_006850/ SPU_021621	MyHC9/10/11
S. purpuratus	SPU_010054	MyHC18A
S. purpuratus	SPU_021119	Sum1/Myod1
S. purpuratus	SPU_006232	Myod2
S. purpuratus	SPU_015983	Myod3
S. purpuratus	SPU_000128	Tropomyosin 1
S. purpuratus	SPU_011034	Tropomyosin 2
B. floridae	estExt_fgenesh2_pg.C_2450027 *	MyHCb1
C. teleta	estExt_fgenesh1_pm.C_1140043 *	MyHCb
S. kowalevskii	XP_002742413.1	MyHCa
S. esculenta	ACD68202.1	MyCvH
L. gigantean	estExt_fgenesh2_pg.C_sca_80337 *	MHCII-1
L. gigantean	fgenesh2_pg.C_sca_98000032 *	MHCII-3
A. irradians	CAA39247.1	MyHC
T. adhaerens	fgeneshTA2_pm.C_scaffold_2000077 *	MyCHb
T. adhaerens	e_gw1.33.84.1 *	MyCHa
A. suum	ADY39976.1	Myosin-3
B. malayi	XP_001899601.1	MyHCb
O. volvulus	AAA29420.1	MyHCb
S. mansoni	AAA29905.1	MyHC
D. pulex	EFX87106.1	MyCH
D. japonica	BAA34954.1	MyHC

*Sequences of these gene models were obtained from Patrick Steinmetz (Steinmetz *et al.*, (2012) Nature 487, 231-4) and are reported below.

>Ta-MHCa *Trichoplax adhaerens* 33:300609-315541e_gw1.33.84.1[Triad1:33303]
MKSSAKELKHLVVEKRAFIDPAVQAQWMAKKYVWIPDDNHGFTAASIKSERGDEATVQLNDGRTTTINREDIQKMNPPKFDKVEDMADLTYLNEASVLHNLRD
RYFSGLIYTYSGLFCVVVNPYKRYPIYTDEVVELYKSKKRHEVPPHIYAITDQAYRCMLLEREDQSILCTGESGAGKTENTKKVIQYLAAVAGAAKEMKDGNDG
GELEAQLLEANPILEAFGNAKTIRNDNSSRFGKFIRINFDAAGFIVGANIETYLLEKSRAIRQAPDERNFHIFYQMLLGATPEMKDDLLLEKIDKYNYMSNQLVVVP
GVDDAEEFQNTLNAIAVMGISNEDTNSSLKILSSLLHMGNLKFDEDKSSDQATLPDNTVAQKVCFLLGVSVTDFTRALLKPRVKVGRDYVVKAQNKDQASFAVQ
ALSKALYKRLFKWLVSRVNRTLDRTKRQGASFIGILDIAGFEIFKLNSFEQLCINFTNEKLQQLFNHTMFILEQEEYRKENIDWKFIDFGLDLQPTIDLIEKPLGILAL
LDEECWFPKATDKTFSDKVEKQHKNHPKYHKPDFRENADFCLIHYAGKVTYCTNQWLMKNMDPLNDNVITLLQQSSDGFVAGLWKDSSNVVGMNATNTDTTF
GSSRTRKGMFRTVGQLYKEQLARLMTVLNNTSPNFVRCIIPNHDKKPGKIEANLVLEQLRCNGVLEGIRICRQGFPNRIAFQEFKQRYEILTPGAIPKGFMDARKAV
QKMTEVLELESNSYRIGLSKLFFRAGTLARLEEERDLKLSSIITGFQAYCRGNLARKMYLRRVQQTNAIRVIQRNVQAYLKLRDWQWWRLYTKVRPLLNVTRQD
DEKRQVEDELKRVREKSEKAQHDLQILEIDYNKVLEEKSILAEQLQTETELCAEAEEMRIRLSIKKNELEELLHDAEVKAAEEEENVNVLTKEKKKLLQSISDLETS
LEEEEAQRQKLQMEKASLDSKIVAFENDLMKEADTNSKLSKDKKFLEDKVAELTSSFGTLEDKHKHLNRQKAKVDSQLQETEDTLRKEQELRTDLEKIKRKLEA
DLRDTNDQLSDATAQVTELQNTLERHSNDLNQANAKIDQEVNARNKAEKKCRDLESQLQEITEDLDAERDTRSKIDKQKRQLNEELESLKNELLDSLDTTAAQQ
DLRNKREEELKHLKKVLEEDTIAHAEAVSLSKKKHNETIEEMNNQIETLKKAKASLEKYKLQADSSLADVKAECSDLHASKTELEKKKKALESQVSDLNGRNTTL
QRKTEDLEQTNSKLQSQLDTTVAQLVQAETQISSLERTSKSLELQIAELQELYEDETKQKQNISMQHRELENKLSHHSELIEELESDKENLARQINTQSTQLADARK
SLQQSEIALEDAENAKRKLAKDLDLCQNTLDDLKNTNTKLEKAKTRLQCEVDDLNVDLEKGKSTISNLEKRQRKFDQQLAEERSKSEMIANDRDTAEKTVRSNE
TTIISLKRELEELRDQHEDSERIRRQQQAEINDLMESKDDAGKNVHELEKAKQTLAAHVEDLKAQLEELEDELQLAEDGKLRLEVNLQAAKSNLDREVSQRDEQI
EEARRSLLKQVRELETEVEDERSQRSQAVSAKKKLEMDVKELESQVELANKSRDDDVRQNKKLQGQVKEMQNELDELRVSREELYDQSRDLDRKFRTAEGDLR
QAQEELANSERARRNAEAERDELIEDSASASSLRQSWTDEKKRLESRISELEDELEDEQTENSSQQDKYRKMQMQMEQLNSDLAVEKSVSQKLEAGKTQLERQN
KDLQAKVSELETSSRAKSKALTQTWESRINNLEEQLESEARERTSLQKNNRRAEKRLKELTIQVEEERRHADEYKEQADKASSRLRNMKRQLDEAEEETSRQTAL
KRKMQRDLDEQMEVSEALTREINALKTRMRLVNYFIAFFLLRFGYIISSDVHIYEI

>Ta-MHCb *Trichoplax adhaerens* 2:7673470-7690114fgeneshTA2_pm.C_scaffold_2000077[Triad1:49924]
MDDAEGFLRQKSQAVTESIDSKKWVWIEDKAEGYLSAKILEVNGETLSVEVNTGQRLTVKRDLTQQMNPTKYDKAEDMAALTYLNEAGVLNNLKQRYFSGMI
YTYSGLFCVAVNPYRRLPIYTDKVVEMYKGKRRAEMPPHIFSITDNAYNEMLQERENQSILITGESGAGKTENTKKVIQYVATVAGTGETKSEKKQNLEDQIVQA
NPLMEAFGNAKTIRNNNSSRFGKFIRVHFGLHGKIAGADVESYLLEKSRVVSQTSEERNYHVFYQILTAADDQMKEKYLVTGKPEDYKFLSEGVARIDAVDDEEE
WHATCDSMKTLRFTDEERGFLIKVVMAILHFGNVKFKQRPREEQAECPDTADAEKVAFLLGIQVADLVRSLLRPRIRVGHEYVQQGRNYEQVVSSVAALSKSLY
DRMFKWLIARINKTLETKFSKNYFIGVLDIAGFEIFQVNLFEQLSINYTNEKLQQFFNHHMFILEQEEYKKENIDWEFIDFGHDLQPCIDLIEKKLGILSILDEESIYPK
ASDKTFIEKLKKNHDGKSPKFKLPKMSSKNKAHFEIEHYAGTVGYTVMGWLEKNKDPLNDSVVDLLRKSTDPIIASLFADHQPEGSRKKGSQFLTVSQLHKEQL
EKLMVNLRNTTPHFVRCIIPNEKKKPGIIEANLVLHQLRCNGVLEGIRICRKGFPNRIIFSEFKQRYAILAPGAIPSGMFMDGRKAAAKLVDALQLEPNEFRMGTTK
VFFRAGVIGRLEESRDEKLYAILSKFQARMRGFLMRKTFQKMQEQRSGLQIIQRNVRKYIVLRNWQWWRLFTKVKPLLNVARSDEELKHMQEELNRTLDKSQKE
ESLRKDLEEQNTKLVEEKQQLFMDLQREQDAYADAEDKIVKLESIKNDMESQIQELNDQLEEEEDNNAQISQVKRKLEAEVENLRTDIEDIEAALQKSDQEKQAK
DDALRSITTELDECQEAVDALTKDKKALEEKELELSNLLEAEEDKSNHLTKVKNKLETSLEELQSHLDSEKKHTSDLDKAKRKLESEAKVANEKINEMEGNVKLA
EETIHKRDNEISKLGQSLEDERTTVAALQKKLREAQSRIDELEEEFDNERVLKLKIEKQKAELLQELDELNEKLDEAGGAHVLQTDLTKKREAEINRLQSEIESRTK
QHGSQVASMKKKHNEIIDELENQVETLQRAKNKLESEKQHLSGDLDELQSNLESLTKAKNSSDKQLRQLDEQYNEASAKVDEQEKIIADLQAALQRLQAENN
ELSEQVEDGESKINSLTKAKKGLESQLEELKRKFEDESQARSSVSNNVKNLQAELEQTQELLDEEQESKAELQRQLTKANADAATWRNKYEIDGAQRVEELEDIK
RKLTAKLTSAEELADEFQMKFSSAEKSKNRLSTEMEDLLLDLERAQNQCAAMEKKQRKVDAQTAEWRNKHDTLTIDLENAQREYREQSTELFRVKGSYQETAE
LVDGLRRENKQLQNELSDLSEQLNSDGRNAAEIEKVRRKLEVEKEEIALLLEEAEASLEQEENKVLRMQLEQSQLKQEFERRIHEKEEEMEGLRKNHQRQLEAVN
NSLGSESKSKSEQIRLKKKLEAEITELELTLEAANKSNADLNRSVKKYQSQVKELQSMIDDEQRVHEDYRDKVQKAERRANDVLGELEDLRASLEQSERVRKQLE
VDQIDLQERVQDLTTSNSAALATRKKLETEIESLHEEIEELEGEAKSAEDKYKRANEQIARLTSEIATEHDEQMKEMEAKLNDSELAGGKASKRHVTKLEARIREL
EADLEAETKQSQEATKQLRKNERRLKELQFSAEEDRKNLERTLAAAEKNNHKMRVLRRQLDEAEEQTTQYQSKVRKLQHEIEECEERAEAAESALAKAKSRARI
ALILFRLYCYCMVLFLHPLSVTKSPIL

>NvMHCa *Nematostella vectensis* 151: 35935-66236estExt_fgenesh1_pg.C_1510004
MKYLVVDRAAISDPVALAEWSSKRLVWIPDEKQGFVGASIKDESKDEYVVEVEGGKKKKVHKDDIQRMNPPKFEKVEDMADLTCLNEASVLHNLKDRYYSGLI
YTYSGLFCVVVNPYKRLPIYTDKVIDMYRGKKRHEVPPHIYAITDTAYRSMLQDRENQSILCTGESGAGKTENTKKVIQYLAAVAGHSYGKVELEKQLLAANPIL
EAFGNAKTIKNDNSSRFGKFIRINFDASGFIAGANIETYLLEKSRAIRQNSNERTFHIFYQMMAGLEAKERGEILLGKPTDYRFLSNGNMPVPGIDDAAELADSIQCL
TDMNMGEDEIFAIYRVVSAVLHFGNLVFKQERNSDQAILPNNEVAQKICHLLSMPVTEFTRAILKPRVKVGRDYVQRAVTKDQADSSVEAIAKALYERLFKWVV
QKINKSMDRTKREGASFIGILDIAGFEIFQFNSFEQMCINYTNEKLQQLFNHTMFILEQEEYRKEGIDWKFIDFGLDLQPCINLLEKPMGIFALCDEECWFPKATDKT
LVEKLIKEHNKHEKFQIPEFRSQAHFSIIHYAGRVDYNCDNWLMKNQDPLNDNITSLMQGSSDTFVVALWKDAEFVSQFANSGESPFGSKVRKGMFRTVSQV
YKDQLARLMLVLNNTNPNFVRCIIPNHEKKAGKIASFLVLDQLRCNGVLEGIRICRQGFPNRMLFQEFKQRYELLTPGVIPKGFMDGRKACEKMLQSLELDSNSY
RIGQSKIFFRAGVLAHLEEERDIKLTEIITIFQAFCRGNIARKQYHKRVQQLSAIRVIQRNCLSYLKLRNWQWWRLFTKVKPLLNVTRQEEELHQREDELRTLREKL
EKLETEYTDLEKKHSQISEEKAIVAEQLESEREVAQETEEMRQRLQTKKNELEELLSDLEGRITEEEENVLALTEDKKKLLKEIQDLEDSLEEEESARQKLHLEKVS
CEAKIKKLEEDLSLVEDTNSKISKEKKTLEEKLNELDQALIDEEEKSKGLAKQKAKQDAMIADLEERLKNEERARQDLDKIRRKLESELAEVSRQLEEAKQQIQEL
EAAVHKKDMELNDLNARYDEEMARRTELEKDKRELENSVEELKEDLEVEKSARAKAEKQRRELSEELESLKSELEESIDTTAAATDMRVKREAELVALKKSVEE
ESAKHENLMSELRQKNSKQIEELTIELESTKKNKNSLEKSKKDLENETSSLTIELKAISQEKQEGDRKIKMLENQMSEANIRLADDEQKVSELTISRNQLQKDLEVQI
SMVESLESKHTALERSKKSLEDTLAETQDSLQEETHHKLMIISKLKDVENEIVSLQEQLEEEEENKSALQKQLQSAQTQCAEMKKVVEERAAALEEADAVKKKLV
REMEALNTTVEEIQGSNAKLEKTRKRLQNEVDDLQLNLDKERSTIANLEKRQRKFDQMMAEEKAISERYAMERDNAEREARQNETKVISLSHELEEYQDKLAES
ERLRKLAQTELEGVMESKDDFGKNVHELEKAKRSLEQQLAEMKQTMEELEDELQATEDAKLRMEVNMQAMKTQFERDLAARDDQNEEKRKALLKQLRELESE
LDEERKLRASAVNARKKQELDLRDLEDQLEAANKVKEDGLRQLKKYQQQLKDIQRDLDDARASRDEIAEHAKENEHKLKQLEADFIQMQEDLSAAERSRKVLE
RERDELAEELATLSGQRGATAEEKRKYEARIAALEEEVEEERTQSELLQEKAKRAMMQMEQMQADLNAERANAQNNESARASLEKQNKDLKQKLADLESSTRS
RHKMAIASLESKLTNMQEQLDLETRERALQSKANRKLERKIKELMLQNEEERRHSDQYREQMEKTSGRMKALKRQVDEAEEESSRLNSSKRKLQRELDDAQAE
NEELNRTVNSLRDRIRGRGGGRSGYTTPPEPRRKARDRPSTVEESDTSMDAAED

>NvMHCb *Nematostella vectensis* 34:382412-407942estExt_fgenesh1_pg.C_340036[Nemve1:240778] corrected with estExt_gwp.C_340061
MEFDPNDPLAILRIDKSQIMAQAKEFESKKWVWIPDPKEGYKAAEVKSTKGDNFLVETNDGQEVEINKNDTEQMNPPKYEKTEDMSNLTYLNEASVVHNLKQR
YFSGLIYTYSGLFCVAINPYRRLPIYTDKIVFAYRGKRKTEAPPHVFCICDNAYQNMLQDRENQSMLITGESGAGKTENTKKVIQYLAHVSGGISKADEEHKKQTG
GSLEDQVIQTNPILEAYGNAKTIRNNNSSRFGKFIRCHFGPQGKLAGADIESYLLEKSRVIYQQEIERNYHIFYQILYGAPKDLHDQLLLESNKTADYAYTAKGCER
ADGIDDVEEWANTELAADTLGFSAEEKLSMYKICAACLHWGNSKFKQRPREEQAEVADPKDLDKTSFLMKLPGADFVKNIVKPRIKVGREYVNQGRNLQQVNY
SIGALTKSLYERMFLWLVDRANQTLMTKDRRAFFIGVLDIAGFEIFQFNSFEQLCINVTNEKLQQFFNHHMFILEQEEYKREGIHWEFIDFGHDLEPTINLIFGKGTG
IFAILEEECIVPKATDQTFLQKLNNTHDGKSAKFGKPKISGKKQVNYHFEIHHYAGTVGYNVDNWLDKNKDPINEAVASLFAKSGDPFISHLWKEYATEGHTRGK
GGSFQTVSAKHKEQLQSLMDTLYSTSPHFVRCIIPNELKKAGVIDTPLVIHQLRCNGVLEGIRICRKGFPNRIPFQEFKQRYQILAPTAVSGQFMDGKKACEKLLAAI
QLEENEYRIGTTKVFFRAGVLGHLEDLRDDRLAKIISMFQAYCKGFLMRKEYRKMCDQRIGISVIQRNVRKYLYLRNWAWWKLYTRVKPLLQVARADEEMKQK
VEQMKEIEEKLGKEEALRKELEEKYTKLVEEKNLLFQDFQREQDACADAEERNAELEGRKADLEAQVKDMLEQLEDEEEASAELSSVKHKLEGEISDLKQDIEEL
DATLKKVEEEGKQKDKNIEQLNEELQQQDEAIAKLQKAKKQVEDERTELEDHLQEEQNKVSHLTKTKLKLESTLDEVNLNLEREKKVRGEVEKVKRKLEGDLK
MTQQTLEETQAEKARTEDEVRKRDANIVELSGKLEDSNNLVESLRKRIRELEARVEELEEELEAERNARSKSERARQELEHELDDLNERLDEQGGATQAQMELNK
KRESDIIKLRKDLEEQALAHEQAVNSMRSKQNQQMQEMQEELDQVKKTKAKLEKEKAQLTNEGDDLAVTVETLQKQKQASEKNNRAISDQLAESKAQNEELRK
NIQELQAIRSRIEAENADVNRQLEEQENKGGQLGKAKKNLEQQLEELKKQLDDEMRAKAEAQARLKNLEADFDAMQENFDEAEDGKADLQKQLSRANTEAQS
WKNKYEQDALAKVEELEDAKRKLAAKLQEMEEALNTAQTKAASAEKTRSRLNSELEDALIDLEKAQTNNANLEKKQKKIDIQINEWRVKLEEVQADLDNSQKE
ARNYSTEMYKIKAAFDEQSEQV
EALKRENKSLASEVNDLADQLGEGGKSVVELEKLRKRLEMEKEELQTALEEAEGALEGEEGKVLKIQLEMTQLKQEFERKLADRDEEIDTLRKNHQRQLDALQA
SLDSEVKSKNELARLRKKFETDCNEMEINLENSMKANQELQKTTKKLQQQVKDLQLMVEEEQRGRDDARESAARAERRSAELAAELEDLRNQLEQADRARRTA
DQERADAVDRLAEVSNQVNNLQQGKRKLEGQVNTMQEELDDAENEAKAADERSKKAAAEFARAQAELVSAQDHATAADRARMSAERIQKDLEMKLEELEAA
GGKALKVQIKKLEQRVKELERELDNEAKRSAESQKLAKKNERRMKEIQFQADEDQKNLARAQENSDRMNNKLKKMRTAVEEAEALAAANLARFRKAQTELEE
AEERVEQLESSLQKARGRARTSGVMSGGRAASRETSYRSSSYRSASSTSLDDE

>Lg-MHCII-3 *Lottia gigantean* 98:484732-496085fgenesh2_pg.C_sca_98000032[Lotgi1:170416]
MAGSYDPNDPDMKYLAVDRKKLMEEQTKAFDGKKACWVPDPQASFLPAEIVSSKGDEITVKITTNNEQRTVKKDQIQQMNPPKYEKIEDMANMTFLNEASVLH
NLRSRYTSGLIYTYSGLFCIAINPYKRLPIYTLSVIAKYRGKRKMEMPPHLFAIADNAYQSMVQERENQSVLITGESGAGKTENTKKVIMYFAQVAAMGQKGDEE
EEKKDEKKGTLEDQIVQCNPVLEAYGNAKTTRNNNSSRFGKFIRIHFGTQGKISGADIEQYLLEKSRVTYCQPAERNYHIFYQLLSNAIPAYHEKLLVSPDPALYSFI
NQGCLTVDSIDDTQEMKDTDKAFDVLGFSEDEKLSYLKCTGAIVHFGEMKFKQRGEQAESDGNAEAEKVAFLLGINPEDLLKGFLKPKIKVGTEFVTQGRTKDQ
VVYSVSALAKSLYDRMFKSMVVRVNKTLDTKAKRQFFIGVLDIAGFEIFDFNSFEQLCINYTNERLQQFFNHHMFVLEQEEYKKEGIEWEFIDFGMDLQACVDLIE
KPLGILSILEEECMFPKASDKSFMEKLYQNHLGKSPNFGKPVGKKVKGEAHFELHHYAGTVPYSITAWLEKNKDPLNETIVELLMHSKEALVQTLFAPSEPAAA
SGGPAKKKKSSAFQTISATHRESLNKLMKNLYSTHPHFVRCIIPNEFKQPGEVDAHLVLHQLQCNGVLEGIRICRKGFPNRLVHSEFKQRYSILAPNAIPDGFVDGK
TVAGKILVALEMDPNEYKLGSTKVFFKAGVLGYLEDLRDERLSSIIAMFQAHIRGYLVRRNYKKLCDKRIGLSVIQKNIRKWLAMRNWLWWKMYVKVKPLLNV
ARAEDEMKQKEEQFEKTKVELEKSEKMKKELEEQNTVLLQQKNDMFLELQTEQDKVVEFEEKVENLIKQKIDFEAQIKEMEERCLDEEDAVGNLTEAKKQLQK
ANDDLKKDLEDMETNLVKAEQEKTSKDNQIKTLNDEIAKQEDMIMKVTKDKKNMEEANKKLSDSLQAEEDKVNHLSKLKSKLEQSLDEFENNLEREKKIRGDV
EKVKRKLEQDLKMTQESVEDLERIKRELEETMRKKDAEINSLNSRVEDEQGLVASLQKKIKELQAIIEELEEELASEKQARSKVEKQRNELSHELEDISDRLDEAG
GATSAQIDINKKRELELLKLRRELEENQLHHDAQIASFRKKQQESANEMSDEIDQLMKIKSKLEKERNHARSEAEELAAQLQHASKNKGMSEKMTKQVENQISEF
HFKLEESTRIVSDLQSSKSKLQMEVSEITRTLEETEHKVSIIIKEKNTIQSQFEETKRCLEEETRIRQKLQSEVRNLTTDIDSLRDQIEEEQDTKCAMQRQLSKANSEA
QSWKSKYESEGLSRMEELEEAKRKLQAKLADAEQNLEAANIKINNLEKVKCRLQTENEDMMIEVERTSTTANILEKKQRSFDKTTSEWQTKFSELQLEIETVQRE
SRALSAELFRVKAKDVESVERMESLRRENKHLADEIRELTDQLSEGGRSVHEIEKIRRRVELEKEELQSALEEAHATLEQEEAKVMRLQLEISQVRQEVDRRVHEK
DEEFENTRRNHQRAIESMQASLEIEIKNKTEAMRIRKKLEHDINELEIALDAANRNRVEAEKTTKKFQLQITEIQTMIEDERRQKEEAREAYNMAERRCGVISCEVE
ELRTSLEQSERSRKTVEVEVTDIHIRISEMTAQFNSINSQKRKLECDIAAMTADIDELHTELRSSEDKCKKAICDVARLTEELRSEQEHCMHIEKIRKSLEATTKELQI
RIDEAEAQGLRGGKRVVQKLEVKIRELQSELETEQMRHSETDKNMRKIERRLKEVLFQADEDKRSQDRLQALVEKLQDKTRTYKKQVEEAEEVASLNLAKFRKV
QSELEDAEERAETAESYSNKLRAKNRSSVSQSRSTSVTRTSVVRMS

>Lg-nmMHCII-1 *Lottia gigantean* 8:4186461-4211712estExt_fgenesh2_pg.C_sca_80337[Lotgi1:229681]
MAEDHLVGLSAQELRYLTVDRSLVNDPSVQAEWAGRKLIWVPDEQNGFVMASIKGERGDQLEVIVDETGQKRTVHRDDVQKMNPPKFSKVEDMAELTCLNEA
SVLHNLKDRYYSGLIYTYSGLFCVVINPYKRLPIYQEKVIELYKGKKRHEVPPHVYAIADTAYRSMLQDREDQSILCTGESGAGKTENTKKVIQYLAYVAASSRST

RSSVSNVSHSTNKELSLGELENQLLQANPILESFGNAKTIKNDNSSRFGKFIRINFDLSGYISGANIESYLLEKSRAVRQAESERSFHVFYQFLNGATPAQRKEFLLED
MSNYRYLSYGNVPVPGVDDSADFQSLAESMGIMGISTDDQSAILRIVSAVLLLGNMQFKQERSSDQATLPDNTVAQKACHLLGLPVTAVTQAFLKPKIKVGRDSV
TKAQTKEQVEFAVEAISKALYERMFKWIVSRINKSLDKTKRQGASFIGILDIAGFEIFKMNSFEQLCINYTNEKLQQLFNHTMFVLEQEEYQKEGIEWKFIDFGLDL
QPTIDLLEKPMGILALLDEECWFPKATDKTFVEKLHKQHTTHPKFIKKDFRADCDFSLVHYAGNVPYQAERWLVKNMDPLNENIVSIMQQASDTFMANLWK
DAEIVGMGAAAAVDTMFGSRTRKGMFRTVSQLYKEQLSKLMSTLRNTNPNFVRCIIPNHEKKAGKINSTLVLDQLRCNGVLEGIRICRQGFPNRIIFQEFRQRYEIL
TPNAIPRGFMDGKKAVEKMIQALELDKNLYRIGQSKIFFRAGVLAHLEEERDLKLTDIIVQFQALARGLLARRNYQKRLQQLNAIRVIQRNCAAYLKLRNWQWW
RLFTKVKPLLSVTGQEEQMVKKEEETKRMKESLEKQQQDLLEFEKRFSQVIEEKNILAEQLQAETELCAEAEEARARLAARKQELEDVMHDMELRVEEEEERYN
QVLEEKKNYQQTLKDLEEQLEEEEQARQKLQLERVSHESKLKKYEEDLALLDDQNQKLNKEKKNLEERLADISLQAVEEEEKAKQLGKLKNKYEAIIADLEERL
RKEQQARQELEKIRRRLETELNDLRDQLNEKRDQVEELQQQLLRREEEVQMSLQKTDEEAGQRSILQKQMRELQNQIQELQEDLETEKESRNKAEKQKRDLNEE
LEALKTELEDSLDTTAAVQDVRQKREHEVSEMKKVVEATQKQHENQIQDMRNKYSAQIEALSEEIENTRKTKGSVEKVKQTLEAENQDLANDLKQVQQAKQES
ERKRKQLDSQLQEAVIKLQELERNKGEVSEKSTKLQSELESVSSQLEQSDTKFLQMTQKASALEAQLADSQDMC
QEETRQKLAAQSKLRQALDEKESLLERLEEEEVKKQLQKQCNDLQQKIVEVKKKAEEDAVNNEALEEFKKKAARDADNYQREVEESKMQADRLDKSRRKLQ
AEVEDLTMELENQRSASTAFEKKSRKFDALLAEEKANAERLAIERDNVERESREKETKILNLTRELDDLQERYDGLERIKQQQQRELDDLVSSKDDVGKNVHELE
KAKRTFQQQVEEQRQQIEELEDELQLAEDAKLRLEVNMQALKAQYERDIQGKEDQVEEGKKSLLRQLREMEAELEEERKQRGAAVNARNKLQGDLNDYEQQV
QMANKVKDDAVKQYKRMVTQMKEVTREVDEIRISRDEMSQAQKDNEKRVKTLEAEVLRLSEDLAASERARRNAESERDELQEEIGGSATSKNSLLDEKRRLEA
RISELEDDVDDERNNCELMAEKARKAQLQVDQIMTDLTSERSVSQKLENQRMQLERQNKEMREKLQELEGQNRARIKATIAALESKVLNLEEQLDLEAKDRATL
SRNNRKLEKRIKELILQGEDERRHADQYKEQVEKANNRVKALKRNIDEAEEEIARLTANKRKLQRELDDQVEQNETHSREITSLRKLRGPGGTTRTTTRTTITSKIL
DDSIDGDEDDKNDTAET

>Bf-MyHCb1 *Branchiostoma floridae* jgi|Brafl1|127185|estExt_fgenesh2_pg.C_2450027
MAGSLLRADPDRYLFGDQKELSKLRSQAFDSKKNCWIPNEKDAFLATEITQTKGDILTVQTEDGKTFTVNKDDTQQMNPPKFEMTEDMANLTYLNEASVLYNLK
QRYFHGLIYTYSGLFCVAINPYRTLPIYSNKVVSMYKGKRRTEMPPHVFMVSDNAYNSMLLGESGAGKTESTKKVIAYFANVAALRKDHVEDKNTKADTKVGT
LEDQVIQTNPVLEAFGNAKTVRNNNSSRFGKFIRVHFQANGKLAGADIETYLLEKSRVTSQQPGERCYHIFYYLLSGRFPELQEKLLLTSDPKFYKFISQGDTRIEG
VDDSMEMKFTDEGFDILGFAPEEKLSIYKLVAGVMYFGNMRFKQRPREDQADIDGTQEAEKISHLLGVPEPELVKSILKPRVKVGNDFVTKGQNMQQCNEAIRAL
AKAIYNRMFSWLVARLNKTLDTRMQRSYFIGVLDIAGFEIFEFNSFEQLCINFTNEKLQQFFNHHMFVLEQEEYKREGIEWEFIDFGLDLEACIALIEKPMGIMSILE
EECMFPKASDDTFKAKLYENHNGKSPNFLKPRVQKEGPHEVHFEVIHYAGVVGYNLYGWLYKNKDPLNESCVSCFAKSHLDLLAALFAEDATQESGGRNPAAG
RKHKKSGAFQTVSAKHREQLNKLMTNLRNTSPHFVRCIIPNEAKAAGEIDSFLVLHQLRCNGVLEGIRICRKGFPNRMIFAEFRQRYQILAPTSIPEGYIEGREASTL
LIEGIDLDPCEYRIGKTKVFFKAGVLGHLEDLRDERLAIIMTMIQARARGLLQRKIFKKMMEQRIGLSIIQRNIRRYLVLRNWAWWRLFTKVKPLLKVVKQEDEMR
AKELEIQQLKEKLAKEEAIRKETEQQNATLSQDKNDLVLQLQAEQDNLADAEERCAALIKTKADYESQIQELRERLEDEEDANAELTSSKRKIEEECDELKHDIED
LEMTLSKHAPHPVCKCTDQPQEIKMATLKVIETNFAKYNTTDLNTTLSSLDKLSAAWMHAPHPVCKCTDQPQEIKMATLKVIETNFAKYNTTDLNTTLSSLDKLS
AAWMVEREKQDYDRKCGQLEAELVDKEDTIDRVTKEKKKLEELNQQTLEDLQAEEDKVNHLNKVKVKLETTLDELEDALDEEKKIRQEVERIKKKLESDLKVA
HDKIADGESKRSLLEEDISKKETAIHVLEAKFEDSQSMVGTLQRRIKDLQARIEELEEELDTERQTRMKVEKHRNDLLRELDELSERLDEAGGASAAQIELN
KKREMELAKIRRELEESTMQHEASVTTLRKRHSDQLTELSEQVESLQRSRTKLDKEKNSLRVELDDVATQLEAVTRIKLTAENAELGRQMDDAEAQVRELSRTK
AIVQQSYEEAKKGLEEETRQRTQLSHQVVQLQHDLDVLREQLDEEQEGRTELQRQLSKANAECASWRTKYETEVIQRLEELEEQKKKLAAKLQIAEEQVEAAQA
KVSSLEKTKNRLAGEVEDLMIDLEKANAAAAALEKKQRLLDKQIAEWKVKCEEITVELDASQKECKSYQIELFKLKGQYDEAMDTIDQLRRENGALKDECRDLS
DQLSDSQKYVHELEKAKKRLDLEKEELLATIEELEASLEGEAAKVSRLQVEIVQVKSDCERRIAEKEEEFETTRKNLLRQLESLQASLEVETKAKNEAIRAKKKLE
ADLNELEVALDNATKANAEAKKTITKLQVTIKELQCRDLSDQLSDSQKYVHELEKAKKRLDLEKEELLATIEELEASLEGEAAKVSRLQVEIVQVKSDCERRIAEK
EEEFETTRKNLLRQLESLQASLEVETKAKNEAIRAKKKLEADLNELEVALDNATKANAEAKKTITKLQVTIKELQVQLDDEIRSREELREQYALLEKRLQLTITEL
EDLRVLYEHSEKVRKAAEADAQDLQDRNNELAAQNASLSAHKRKVDAELQALQVELDETVSELKAADERAKKASAEAAHLADELRAEQETCLQLDKTKKNLE
VTLKDLQLRLDEAEAIALKGGKRLIQKLEARVRELEQELENEQRRHSETQKNMRKNERRLKELSFQAEEDRKTQERMQELIEKLQLKIKSYKRQVEEIEEQATINL
SKFRKTQHELEDAEERAEIAEGSLNKLRARNRGTVSPATRITTPSTASRSNGPYTDKTFETSSYPRRYYSSHGSTPSTPPRDLEDGDRTPPRRPSISQLSTGSTGTPHS
SNTNNTPEAEVDGGEEEE

>Ct-MyHCb *Capitella teleta* jgi|Capca1|157039|estExt_Genewise1.C_1140043
MEEMTIKKDNIHQMNPPKFEQTSDMANLTYLNEAAVLYNLKARYGAGLIYTYSGLFCVVINPYRRLPIYTKQVIDKFQGKRRSEMPPHLFSVADNAYRNMLQDR
ENQSMLITGESGAGKTENTKKVISYFASVAAGQQKAAEAAGTAEKGKATLEDAIVQANPALEAYGNAKTIRNNNSSRFGKFIRIHFGTNGKIAGADIETYLLEKSR
VTFQQSEERNYHIFYQLLSGKFPEYCDKLLVTPDAGLYHFINQGCLTVDNMDDVEEMKIVDHGFDVLGFTKEEKMSLYRCTCSVMHFGEMRFKQRPRQEQAEA
DGSSEAEKVAFLLAVNAGDLLKALLTPKVKVGNEYVTKGQTKHQVVYAVAALAKALYARMFGWLVARVNQTLETKHKKQYFIGVLDIAGFEIFVYNTFEQLCI
NYTNERLQQFFNHHMFVLEQEEYKKEGIHWEFIDFGMDLQACIELIEKPMGILSILEEECMFPKASDKTFLTKLFDNHMGKSANFGKPKPSKGNAEFAPHFELYHY
AGTVGYNTNGWLDKNKDPINDTVVQLLQASKEPLVSMFFAEPKEDANAPKKKKKGGGFQTISATHRESLHKLMANLKTTHPHFVRCIIPNELKQPGLIDAKLVL
HQLQCNGVLEGIRICRKGFPNRIIYSEFKQRYSILAPNAIPQGFVDGKVVSEKVLTALQLDDNDYRLGHTKVFFRAGVLGTLEDMRDERLAKIIANFQAFIRGYLIR
RNYKTLQDQRLGLSVIQRNIRRWLTLRNWQWWKLYARVKPLLSIARQEDEMKKIEEDLIKTKEELEKTERQKKELEEQNVGLLQSKNDLFLQLQAEQDNLSDAE
ERVIQLISQKGDYEERLKELEERLSDEEGTSEELESAKAKLENECESLRADIENLELNLQKAEQDKQSKDNQIKTLNEEMARQDEQIAKMQKEKKNTDEVLKKTQ
EDLQAEEDKCNHLNKLKQKLEQSIDELEDNLEREKKHRADVEKTKRKLESDLKMTQETVDDLERVKKDLEDGMRKKDGEINSLNSRLEDEQSLVAQLQKKIKEL
MARIEELEEELEAERAARSKVEKQRAGLQAELDELSERLEEAGGATQAQIDVNKKREAELQKLRRDLEEATIQNEQNTVALRKKQQDAVNELSDQVEQLNKIKQ
KVEKERNQIRNEVEDLQSQVEHVTKSKSAADKLAKQLEHQLSDANSKLDDSARQISEMQGTMGRSQSEAADLSRQMEEAESQISQLTKAKQALQKQLEEAKSQL
EDESKMRAKLQGENRNLNTDLDQLRDQLEEEQEGRGDLQRLLTKANGEIQVWRQKFESGEGGVRSEEMDDMKRKMNAKLQELESQLEGALSKASSLDKAKNR
LQGEIEDLMIEVERSQSIANQAEKRQRAFDKTIDEWKRKVADLQSELETSQKDGRNTAAEVYRLKAQIEEGSDTVNALRRENKNLADEIHDLTDQLGEGGRSTHE
LEKARKRLEMEKEELQSALEEAEAALEQEEVKVQRSQLEISAIRQDIDRRLAEKDEEFENTRKNHQRALDSMQASLEAEARGKAEALRMKKKLEQDINELEVALD
GANRGRAEAEKNIKKFQQQMMEIQSAIEDEQRARDEAREQFQSAERRANMLAGELEELRTQLEAAERARKAAEGELHEASDRVSELSISNASLTATKRKLETDIQ
AMQTDLEDQSGELKAAEEHAKKAMADAARIAEELRQEQDHAGQIEKMRRGMESQVKDLQARLDEAEAAALKGGKRMIQKLEQRVRELEVELDNEQKRHQET
QKNMRKQDRRLKELAFQSDEDRKNQERMQDMIDKLQQKIKTYKRQVEEAEEIAAINLAKYRKVQHELEEAEERADMSENTLAKLRAKNRSSVSATRTTSSSPYG
YGYSSSFSSTNRNIRSSTFGGSQNSLNNLTFRNGSDPVTDDTY
