# Supplementary figures and images for: Myogenesis in the sea urchin embryo: the molecular fingerprint of the myoblast precursors
Source: EvoDevo. 2013 Dec 2;4:33. doi: 10.1186/2041-9139-4-33 (PMC4175510; doi:10.1186/2041-9139-4-33)

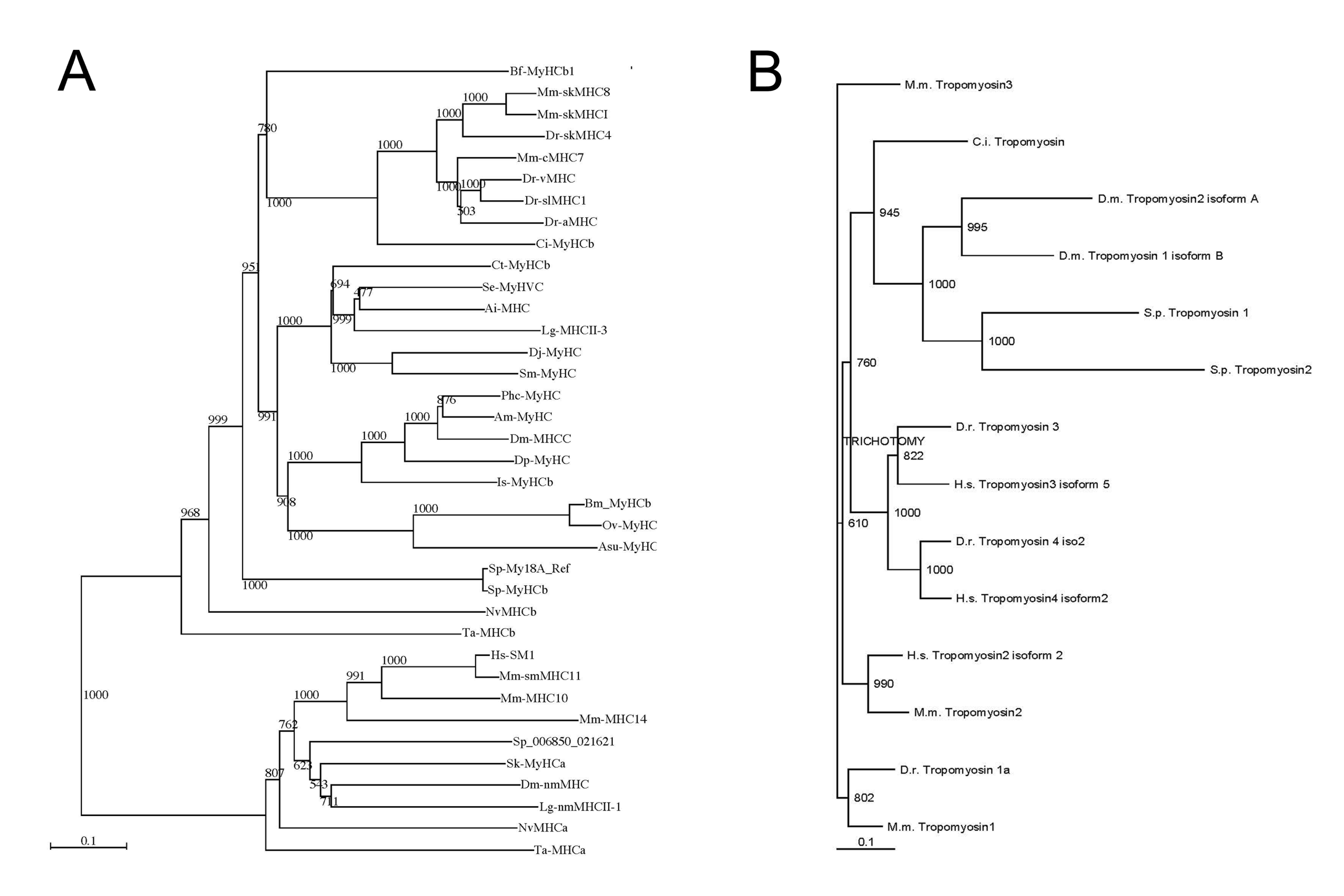

Supplement: Additional file 3: Figure S1 — Phylogenetic tree of MHC and Tropomyosin sequences. Neighbor-joining trees of MHC (A) and Tropomyosin (B) proteins from cnidarians, ecdysozoans, lophotrohozoans, placozoans, hemichordates, cephalochordates, urochordates, vertebrates and the sea urchin S. purpuratus. In panel A, Sp-My18A and Sp-MyHCb are the same protein, annotated twice. Similarly, Sp-006850 and Sp-021621 are different domains of the same protein. The trees were generated from the alignment of the amino acid sequences of the MHC proteins using CLUSTAL X and TreeView. Numbers indicate bootstrap support for given nodes. Maximum parsimony methods also confirmed all group nodes. All named proteins are appended with the species designation (one letter for the genus, one for the species). Accession numbers and sequences used are provided in Additional file 2: Table S2. [file 2041-9139-4-33-S3.tiff]

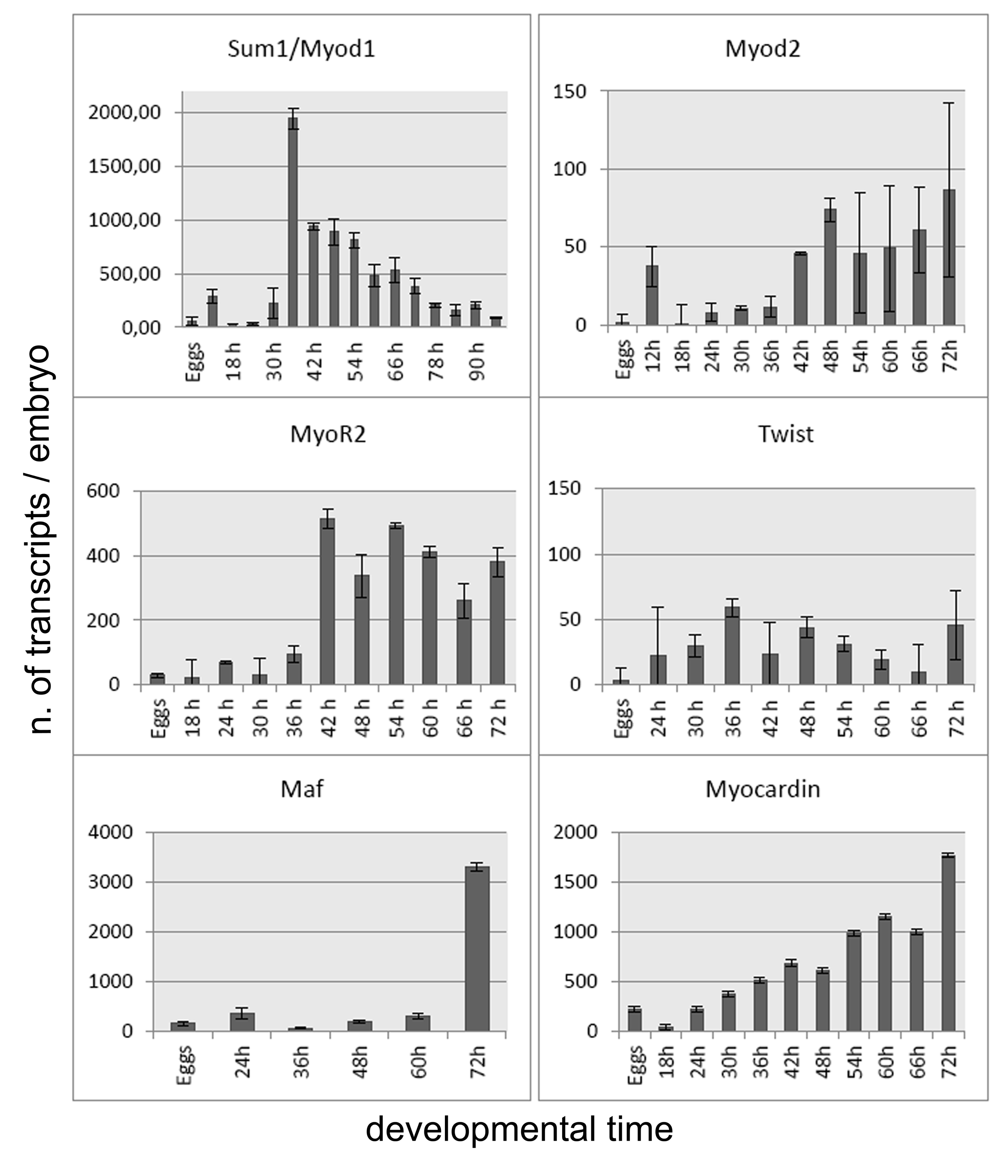

Supplement: Additional file 7: Figure S5 — MyoD1/Sum1, MyoD2, MyoR2, MAF, Twist and Myocardin temporal expression profile during sea urchin embryogenesis. Graphs show the temporal expression profile revealed by qPCR and expressed in number of molecules per embryo. Average calculations over the various measurements ± standard deviations per individual time points of development are reported as columns with error bars. Two MyoRs were identified in the sea urchin genome; MyoR2 and MyoR4. From these two, only MyoR2, which in the current work is referred to as MyoR, showed significant expression as measured by qPCR. [file 2041-9139-4-33-S7.tiff]

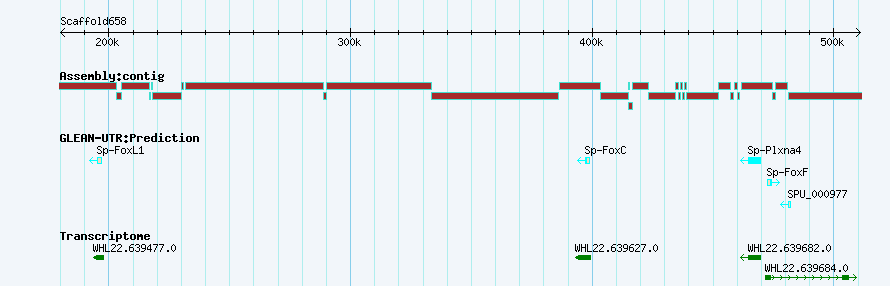

Supplement: Additional file 12: Figure S10 — Genomic organization of FoxL1, FoxC and FoxF. An S. purpuratus genomic scaffold (approximately 300 Kbp) (http://www.spbase.org) is shown. This genomic region includes both predicted sequences of FoxL1, FoxC and FoxF (light blue) and transcripts (green). [file 2041-9139-4-33-S12.png]
